# Assessment of Fluid Responsiveness via Central Venous Ultrasound Measurement: A Network Meta-Analysis

**DOI:** 10.3390/jcm14020492

**Published:** 2025-01-14

**Authors:** Levan B. Berikashvili, Ivan V. Kuznetsov, Mikhail Ya. Yadgarov, Pavel V. Ryzhkov, Petr A. Polyakov, Andrey G. Yavorovskiy, Alexey A. Yakovlev, Andrey V. Grechko, Valery V. Likhvantsev

**Affiliations:** 1 Federal Research and Clinical Center of Intensive Care Medicine and Rehabilitology, Moscow 107031, Russiaikuznecov@fnkcrr.ru (I.V.K.); petrpoljakov01@gmail.com (P.A.P.); avgrechko@fnkcrr.ru (A.V.G.); 2 Department of Anesthesiology and Intensive Care, First Moscow State Medical University, Moscow 115409, Russia

**Keywords:** ultrasonography, fluid therapy, hemodynamics, network meta-analysis, venous pressure, diagnostic techniques and procedures

## Abstract

**Background:** Ultrasonographic assessment of the diameters of various veins and their indices are among the most applied diagnostic tools for evaluating fluid responsiveness in clinical practice. Despite their widespread use, there is no definitive answer on which is preferable. Our study aimed to investigate the diagnostic accuracy of different venous diameters and their indices to assess fluid responsiveness. **Methods:** We conducted a systematic review and network meta-analysis, analyzing prospective studies evaluating the diagnostic accuracy of venous diameters (inferior vena cava [IVC], internal jugular vein [IJV], superior vena cava, and subclavian vena) and their indices for fluid responsiveness. Electronic databases were searched from inception until March 2024; this search was supplemented by snowballing methods. The risk of bias was evaluated with QUADAS-2, and evidence certainty was assessed using the GRADE approach. Nine prospective cohort studies (560 patients) were included. **Results:** The network meta-analysis revealed that the ΔCaval index exhibited a significant performance advantage over other “venous” test parameters. The caval index significantly outperformed IJV min/max and IVCmax. IJV index and IVCmin significantly outperformed IJVmin/max. The caval index was comparable to the IJV index. The caval index was comparable during mechanical ventilation and spontaneous breathing. **Conclusions:** In this meta-analysis, the ΔCaval index test showed higher diagnostic accuracy for fluid responsiveness compared with other venous tests. Caval and jugular indices displayed similar accuracy, and caval indices were consistent under mechanical ventilation and spontaneous breathing. Indices generally outperformed absolute values, except for IVCmin, which equaled the caval index in efficacy. This study was registered on the International Platform for Registered Protocols for Systematic Reviews and Meta-Analyses: INPLASY202430104.

## 1. Introduction

Hypotension is a major concern in medical fields such as anesthesiology and critical care. According to the 2022 clinical guidelines, the primary step in evaluating hypotension is assessing fluid responsiveness [1]. Despite the availability of various tests for this purpose, there is no consensus on which test offers the highest diagnostic accuracy [2,3,4,5,6]. Consequently, no specific method is universally recommended for assessing fluid responsiveness.

Ultrasonographic measurement of the diameters of certain veins, such as the inferior vena cava, jugular, femoral, or subclavian veins, is a common method for assessing fluid responsiveness. The tests mentioned above are noninvasive, straightforward to perform, and enable quick conclusion delivery [7,8]. The diameters of these veins, especially the inferior vena cava, serve as indicators for evaluating venous return effectiveness [9]. Additionally, these vein sizes are affected by intrathoracic pressure and respiration type, causing diameter variations during inhalation and exhalation. Consequently, various venous indices have been developed, including measurements during both inhalation and exhalation phases and considering respiration patterns [10]. Meta-analysis data suggest that measuring the indices of the inferior vena cava and internal jugular vein provides significant diagnostic accuracy and is promising for the initial management of hypotension [11,12,13,14,15]. However, there are no network meta-analyses comparing different methods of assessing fluid responsiveness, particularly evaluations of various veins and their indices.

Understanding the assessment of fluid responsiveness requires a discussion of the physiology of intravascular volume distribution and the role of capacitance vessels. The venous system, particularly the large veins, acts as a reservoir for blood, holding approximately 70% of the total blood volume [16]. Capacitance vessels, which include veins and venules, have high compliance and can accommodate changes in blood volume with minimal changes in pressure [16]. Factors such as intrathoracic and intra-abdominal pressures significantly influence the volume and pressure within these vessels [17]. Elevated intra-abdominal pressure, for instance, can compress intra-abdominal veins, thereby affecting venous return and cardiac preload [18]. Understanding these physiological principles is crucial for interpreting changes in venous diameters and assessing fluid responsiveness accurately.

Another important consideration is the difference between spontaneous and mechanically ventilated patients. In spontaneous breathing, negative intrathoracic pressure generated during inhalation increases venous return, while positive pressure during exhalation decreases it [19]. Conversely, mechanically ventilated patients experience positive intrathoracic pressure during inhalation, which can decrease venous return and complicate the interpretation of venous diameter measurements [20]. These differences necessitate distinct approaches to assessing fluid responsiveness in spontaneous versus mechanically ventilated patients, as the respiratory patterns and pressures exerted on the thoracic cavity differ significantly.

This research represents a network meta-analysis aimed at exploring the distinctions in diagnostic accuracy among various fluid responsiveness assessment tests, evaluating different venous diameters and their indices.

## 2. Materials and Methods

This systematic review and network meta-analysis adhered to the guidelines outlined in the Preferred Reporting Items for Systematic Reviews and Meta-Analyses incorporating Network Meta-analyses (PRISMA-NMA) Extension Statement [21] and followed the methodological recommendations provided in the Cochrane Handbook for Systematic Reviews of Interventions [22]. The study protocol was prospectively registered with the International Platform of Registered Systematic Review and Meta-analysis Protocols (INPLASY) under registration number INPLASY202430104 (https://inplasy.com/inplasy-2024-3-0104/). A detailed PRISMA-NMA checklist is available in Supplementary Table S1.

### 2.1. Search Strategy

A comprehensive systematic search was performed to identify relevant studies published from database inception through March 2024. The search covered major databases, including PubMed (MEDLINE) and the Cochrane Central Register of Controlled Trials (CENTRAL). Two independent investigators conducted the search to ensure rigor and reproducibility. Forward and backward citation tracking was additionally employed using the Litmaps tool [23]. This approach enabled the identification of additional studies through citation analysis, authorship patterns, and content similarity based on abstracts and titles, utilizing AI-based algorithms. No language restrictions were applied. The complete search strategy, including detailed queries, is provided in Supplementary S1.

### 2.2. Eligibility Criteria and Study Selection

Following the automatic removal of duplicate records, two independent reviewers screened the remaining studies for eligibility based on titles and abstracts, applying the PICOS criteria (Supplementary S2). We focused on prospective cohort studies evaluating the diagnostic accuracy of ultrasound measurements of central veins—including the inferior vena cava (IVC), superior vena cava, subclavian vein, and jugular vein—as index tests (or test methods) for assessing fluid responsiveness. Fluid responsiveness was defined using the fluid challenge (FC) method, regarded as the ‘gold standard’.

Studies were excluded if they met any of the following criteria: (1) review articles, case reports, or letters to the editor; (2) use of an inappropriate reference standard; (3) absence of data relevant for network meta-analysis; or (4) assessment of non-standard test parameters.

Discrepancies between reviewers were resolved through discussion and, when necessary, consultation with a supervisor until consensus was achieved.

### 2.3. Outcome Measures and Data Extraction

A dedicated data extraction form was specifically designed for this review. Three independent investigators utilized this form to evaluate the full-text manuscripts, along with any supplemental or additional materials associated with the included studies. Data extraction focused on the following domains: (1) general study and patient characteristics, including the first author, study setting, sample size, mean age, sex distribution, body mass index (BMI), APACHE II score, baseline central venous pressure (CVP), and type of fluid administered; (2) details of the index test and reference standard, such as the method, parameter, and cut-off criteria; and (3) outcome measures, including the reported area under the receiver operating characteristic (AUROC) curve and the number of responders versus non-responders.

Following independent data extraction, discrepancies among reviewers were resolved through discussion to achieve consensus. For studies presenting continuous data as medians, interquartile ranges, or 95% confidence intervals, established statistical methods were employed to estimate the mean and standard deviation (SD). These calculations adhered to approaches described by Luo et al. [24] and Wan et al. [25], as well as methodological recommendations outlined in the Cochrane Handbook [22].

In the absence of data on the SDs or 95% confidence intervals (CIs) for the AUROCs, we utilized multiple imputation implemented in IBM SPSS Statistics (version 29.0) with the fully conditional specification (FCS) method. Imputed values were pooled using Rubin’s rules. Multiple imputation was applied exclusively to AUROC data. For all other variables, complete case analysis was performed without imputation.

The primary outcome of this meta-analysis was the AUROC for the test method.

### 2.4. Data Analysis and Synthesis

A frequentist random-effects network meta-analysis (NMA) was performed using the CINeMA (Confidence in Network Meta-Analysis) framework [26] and CINeMA software v. 2.0.0 [27]. Additionally, a Bayesian random-effects NMA was conducted employing the ROB-MEN approach [28] and the MetaInsight web-based application [29]. Studies were eligible for inclusion in the NMA if they compared two or more ultrasound test parameters. The mean difference (MD), along with 95% confidence intervals (CIs), was calculated for the area under the receiver operating characteristic curve (AUROC).

The NMA findings were visualized through network plots, league tables, contribution matrices, and forest plots. The surface under the cumulative ranking curve (SUCRA) values were computed to estimate the probability of each test parameter being the most effective for evaluating fluid responsiveness [30]. Statistical heterogeneity was explored using 95% prediction intervals to assess the range of expected effects in future studies. Between-study heterogeneity was evaluated through Bayesian NMA with τ^2^ estimation, where τ^2^ values exceeding the predefined clinically relevant effect size (MD ≥ 0.1) were considered indicative of substantial heterogeneity. Incoherence within the network was assessed via the CINeMA framework.

In order to explore potential sources of heterogeneity, a meta-regression analysis was conducted using a restricted maximum likelihood (REML) random-effects model. This analysis evaluated whether variations in AUROC metrics were influenced by covariates, including the gold standard and test criterion, proportion of responders, mean age, and sex distribution [31].

All covariates were tested in a univariate model using STATA 17.0 (StataCorp, College Station, TX, USA) software. Statistical significance was set at 0.05 for hypothesis testing.

### 2.5. Internal Validity and Risk of Bias Assessment

The internal validity and risk of bias were evaluated independently by two reviewers utilizing the ‘Quality Assessment of Diagnostic Accuracy Studies’ (QUADAS-2) tool [32] (Supplementary Table S2). Potential publication bias and small-study effects were analyzed through Bayesian network meta-regression. The overall certainty of evidence was appraised using the GRADE framework, which was incorporated into the CINeMA approach.

### 2.6. Subgroup and Sensitivity Analysis

Sensitivity analyses were performed, including only studies with a low to moderate risk of bias. Subgroup analyses were also conducted to evaluate the Caval index test parameter (spontaneous breathing versus mechanical ventilation [MV]).

## 3. Results

### 3.1. Study Characteristics

**Figure 1:**
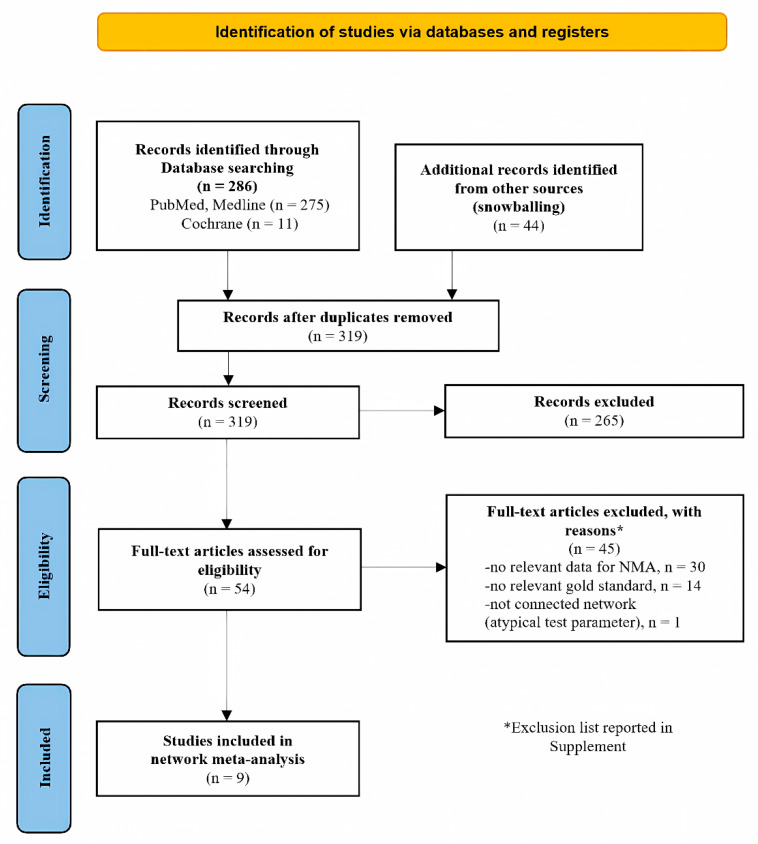
PRISMA flow diagram for study selection.

The initial database and supplemental search yielded 330 articles. After screening titles and abstracts, 54 articles were selected for full-text assessment. Upon detailed evaluation, 45 studies were excluded (Supplementary Table S3). Consequently, this systematic review and meta-analysis included nine prospective cohort studies published between 2013 and 2022 [33,34,35,36,37,38,39,40,41]. The study selection process is presented in the flowchart (Figure 1).

This meta-analysis analyzed data from 560 patients. Among the included studies, three focused on shock [34], major trauma [35] and abdominal surgery [41], while others investigated non-classified ICU patients [33,36,37,38,39,40]. The mean age of the participants ranged from 56.8 to 68.4 years (SD 3.9). Crystalloid solutions were used for FC in five studies [33,34,36,38,41]. Nine parameters were used as tests, and six were used as the ‘gold standard’ parameters for assessing fluid responsiveness (Table 1 and Table S4). The volume of infusion for FC varied and included 500 mL and 5–7 mL/kg. The percentage of responders varied from 39% to 60%. The characteristics of the studies included in the meta-analysis are summarized in Table 1 and Table S4.

### 3.2. Network Meta-Analysis

**Figure 2:**
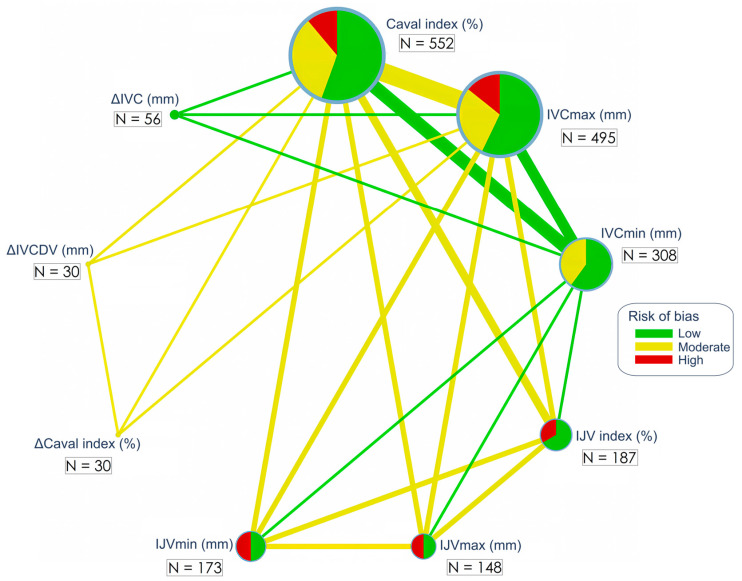
Network plot for ultrasound test parameters used for fluid responsiveness assessment. Node size represents sample size, while node color indicates the risk of bias (green—low risk, yellow—moderate, and red—high risk). Edge width corresponds to the number of studies, and edge color reflects the average risk of bias. The total number of patients for each node is presented.

The network plot is presented in Figure 2.

The data used in the NMA, including the NMA characteristics, league tables and contribution matrices, are comprehensively detailed in Tables S5–S13 and Figure S3.

The pooled AUROC for the test parameters varied from 0.5 to 0.88 (Table S6).

**Figure 3:**
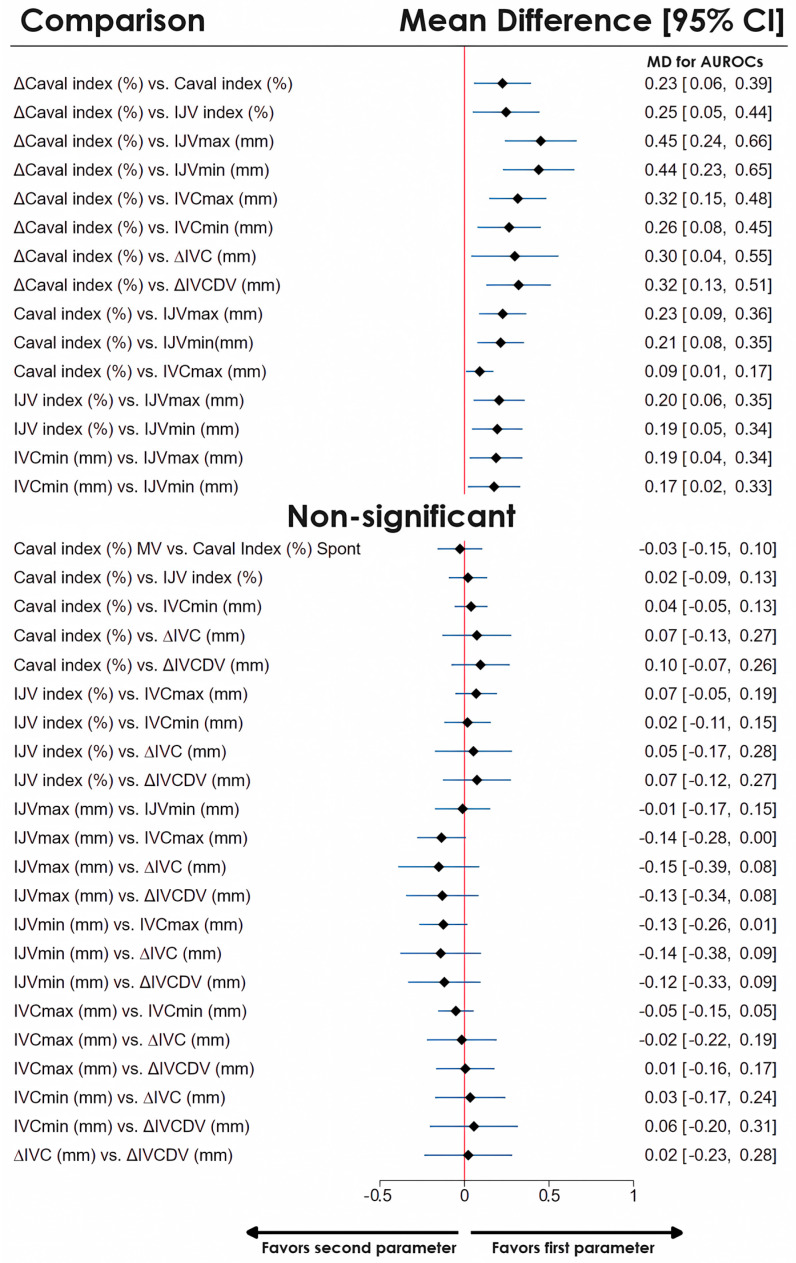
Network meta-analysis summary forest plot comparing the efficacy of various ultrasound test parameters used for assessing fluid responsiveness. The mean differences with 95% CIs for the AUROCs are presented.

ΔCaval index exhibited a significant performance advantage over other test parameters (MDs from 0.23 to 0.45, Figure 3, Table S7). The caval index significantly outperformed the internal jugular vein diameter (IJV) max (MD 0.23, 95% CI 0.09–0.36), IJVmin (MD 0.21, 95% CI 0.08–0.35) and IVC max (MD 0.09, 95% CI 0.01–0.17). Moreover, the IJV index and IVCmin significantly outperformed the IJVmin and IJVmax (with MDs ranging from 0.17 to 0.20, Figure 3, Table S7). Notably, the caval index was comparable to the IJV index (MDs from −0.09 to 0.13, Figure 3, Table S7). Other comparisons showed nonsignificant results.

**Figure 4:**
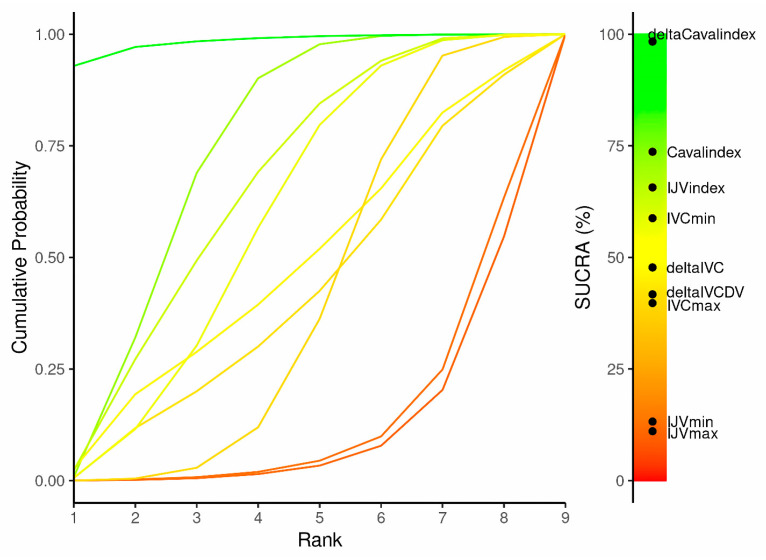
Litmus Rank-O-Gram. Higher surface under the cumulative ranking curve (SUCRA) values and cumulative ranking curves closer to the top left indicate better performance in assessing fluid responsiveness.

The SUCRA analysis revealed that the ΔCaval index (score 98.4) exhibited the greatest potential for assessing fluid responsiveness. The following, in descending order of diagnostic capability, were Caval index (score 73.7), IJV index (score 65.7), and IVCmin (score 58.8); the SUCRA score for the other parameters did not exceed 50 (Figure 4 and Figure S1).

### 3.3. Sensitivity Analysis

The main results (with the exception of IVCmin vs. IJVmin/max comparisons) were confirmed when considering studies with a low-moderate risk of bias (Table S8).

The Caval index MV and Caval index for spontaneous breathing showed comparable AUROC values (MD −0.03, 95% CI −0.15–0.10) (Table S8).

### 3.4. Meta-Regression

Univariate meta-regression revealed no statistically significant covariates for the association between the Caval index and fluid responsiveness defined by FC (Table S8).

### 3.5. Risk of Bias and GRADE Assessment

Evaluation of the risk of bias across the nine included studies identified a low risk in five studies, a moderate risk in three studies, and a high risk in one study (Table S14). The primary sources of bias were related to the implementation and interpretation of the test method, as well as inconsistencies in the application of gold standard parameters.

The risk of bias bar chart is presented in Figure S2. We did not observe publication bias or small-study effects in this NMA. The between-study variance was not significant; however, significant incoherence was observed (Table S11).

The CINeMA ratings can be found in Figure S4. The certainty of the evidence for the studied comparisons varied from ‘very low’ to ‘moderate’. The level of evidence supporting the equivalence of the Caval index MV and Caval index for spontaneous breathing was categorized as ‘moderate’.

## 4. Discussion

### 4.1. Key Findings

In a comparative analysis of “venous” tests to diagnose fluid responsiveness, we revealed that the ΔCaval index has superior diagnostic accuracy in determining fluid responsiveness compared with IVCmax, IVCmin, IJVindex, IJVmax, and IJVmin. The diagnostic quality of the Caval index does not significantly differ from the IVCmin, but it exceeds the IVCmax. 

Furthermore, the IJV index diagnoses fluid responsiveness better than IJVmax and IJVmin. When assessing the IJV, the absolute results are less informative, and it is better to rely on the index. In addition, the Caval index does not significantly differ from the IJV index in terms of fluid responsiveness assessment.

Additionally, this study demonstrated that the use of indices is preferable to the use of absolute values, with the exception of IVCmin. Among all absolute measures, IVCmin is more informative than IVCmax, IJVmax, and IJVmin.

Moreover, we showed that the Caval index during mechanical ventilation does not significantly differ from the Caval index during spontaneous breathing.

### 4.2. Relationship with Previous Studies

Despite the significant number of studies and meta-analyses, the data regarding the diagnostic accuracy of various “venous” tests are different, and there are no network meta-analyses on the subject.

Taccheri T. et al. demonstrated that the ∆Caval index surpasses the IVCmax and the ∆IVC when expressed in absolute terms [38]. In our meta-analysis, we not only confirmed this result but also showed that the ∆Caval index surpasses other ‘venous’ tests in diagnostic qualities for determining fluid responsiveness. Kim D.W. and colleagues came to a conclusion similar to ours: in a comparative analysis of the ultrasonographic variation of the IVC, there was no difference between patients on mechanical ventilation and those breathing spontaneously [15].

This work represents the network meta-analysis on this topic. Network meta-analysis exploits all available direct and indirect evidence, and empirical studies have suggested that it yields more precise estimates of intervention effects than a single direct or indirect estimate [42,43]. In addition, NMA helped us to provide information on the ΔCaval index vs. IVCmin, ΔCaval index vs. IJVindex and other comparisons that have never been evaluated within individual trials.

For the first time, we demonstrated that the Caval index does not significantly differ from the IJV index in terms of fluid responsiveness assessment, which could be an extremely important conclusion in terms of practical application.

Ismail et al. demonstrated that the Caval index and IVCmin possess good diagnostic accuracy as tests for determining fluid responsiveness [34]. In our work, we concluded that the diagnostic accuracy of the Caval index and IVCmin is comparable.

### 4.3. Significance of the Study Findings

The significance of this meta-analysis lies in the following aspects:

First, we demonstrated that the ∆Caval index surpasses other “venous” tests in assessing fluid responsiveness. This finding indicates that dynamic tests, which assess changes in venous parameters, are superior to static ones, which measure a single parameter at one point in time. From a physiological standpoint, dynamic tests are expected to perform better because they account for variations in intrathoracic pressure and venous return, providing a more accurate reflection of a patient’s fluid status [17,18]. This evidence supports the preference for dynamic tests in clinical practice.

Second, we found that the diagnostic accuracy of caval indices during mechanical ventilation and spontaneous breathing is comparable. This finding holds considerable clinical significance regarding the application of this test, suggesting its utility across a varied patient cohort. Nevertheless, further research is needed to confirm this conclusion.

Third, the Caval index is comparable to the IJV index in terms of fluid responsiveness assessment. This result is highly valuable for clinicians, as it allows them to choose between both methods based on their preferences and expertise. Additionally, the internal jugular vein is much easier to assess using ultrasound, unlike the inferior vena cava, which may have serious limitations for visualization, such as abdominal obesity or the inability to assess it intraoperatively during abdominal surgeries [44]. The internal jugular vein can also be evaluated at the time of catheterization, which may be relevant for the routine application of this test.

Finally, we found that there were no significant differences in the diagnostic accuracy between the IVCmin and the Caval index. This conclusion confirms that in clinical practice, from a diagnostic standpoint, the extent to which the inferior vena cava collapses is more important than how much it stretches. This can also be useful in clinical practice, as measuring the minimum diameter of the inferior vena cava is easier and faster than calculating the Caval index, which includes several parameters in addition to the minimum diameter of the inferior vena cava.

Based on the obtained data, the ∆Caval index emerges as the preferred clinical indicator for assessing the condition of the inferior vena cava, as it reliably reflects fluid responsiveness. While other parameters may be easier to evaluate, their diagnostic accuracy is comparatively lower, limiting their clinical utility.

### 4.4. Strengths and Limitations

The strengths of this work are as follows. First, since this meta-analysis is network-based, it allows not only direct but also indirect comparisons of different methods, which helps improve the quality of evidence. Nevertheless, this approach does not account for studies that evaluated only a single method. Additionally, all studies included in the meta-analysis were prospective, and most of them had a low or moderate risk of bias, which also increases the quality of the evidence. Moreover, the sensitivity analysis for studies with a low to moderate risk of bias confirmed nearly all the results. Furthermore, in this NMA, we did not identify any publication bias or small-study effects.

The inability to use a single gold standard and the small number of direct comparisons for many test parameters are the disadvantages of this meta-analysis. The presence of diverse patient cohorts can be considered both an advantage and a disadvantage. Additionally, the small sample sizes in most primary studies represent a significant limitation, impacting both individual studies and the findings of the network meta-analysis.

### 4.5. Future Studies and Prospects

For a thorough assessment of the diagnostic accuracy of “venous” tests, additional research with rigorous methodological standards is essential. This entails designing research with robust sample sizes, standardized methodologies and unified criteria for evaluating fluid responsiveness. Such meticulous approaches will enable a more accurate assessment of the efficacy and reliability of “venous” tests across diverse patient populations and clinical settings.

Moreover, more detailed patient stratification is required, taking into account factors such as intra-abdominal hypertension, structural abnormalities of the right heart valves, congestion in the systemic and pulmonary circulations, and other conditions that may affect the diameter of the inferior vena cava.

## 5. Conclusions

In this meta-analysis, the ΔCaval index test demonstrated superior diagnostic precision in assessing fluid responsiveness relative to other ‘venous’ tests. Equivalency was noted between the caval and jugular indices in terms of diagnostic accuracy. Similarly, the caval index was comparable during both mechanical ventilation and spontaneous breathing. In addition, the use of venous indices was preferable to the use of absolute values, with the exception of IVCmin, which was equivalent to the caval index.

## Figures and Tables

**Table 1:** Characteristics and description of the nine trials included in the meta-analysis, which applied a fluid challenge and assessed ultrasonographic test parameters.

№	Study	Setting	Test Parameters	Gold Standard Parameter	Type of Fluid Used	Sample Size
1	Baker A.K., 2013 [40]	Non-classified ICU patients	Caval index (%) MV, IVCmin (mm)	∆SV (%)	Colloid	25
2	Airapetian N., 2015 [33]	Non-classified ICU patients	Caval index (%) Spont., IVCmax (mm)	∆CO (%)	Crystalloid	59
3	Ma G.G., 2018 [39]	Non-classified ICU patients	Caval index (%) MV, IJV index (%), IJVmax (mm), IJVmin (mm), IVCmax (mm), IVCmin (mm)	∆SV (%)	Colloid	70
4	Bortolotti P., 2018 [37]	Non-classified ICU patients	Caval index (%) Spont., IVCmax (mm), IVCmin (mm)	ΔVTI (%)	Colloid	55
5	Doucet J.J., 2020 [35]	Major trauma patients	Caval index (%) Spont., IJV index (%), IJVmax (mm), IJVmin (mm) IVCmax (mm)	IVCD (mm)	Mixed	77–123 (depends on test parameter)
6	Taccheri T., 2021 [38]	Non-classified ICU patients	Caval index (%) MV, IVCmax (mm), ΔCaval index (%), ΔIVCDV (mm)	∆CI (%)	Crystalloid	30
7	Ismail M., 2022 [34]	Shock	Caval index (%) Spont., IVCmax (mm), IVCmin (mm)	MABP (mmHg)	Crystalloid	102
8	Elsaeed A., 2022 [36]	Non-classified ICU patients	Caval index (%) Spont., IJV index (%)	∆CI (%)	Crystalloid	40
9	Ma Q., 2022 [41]	Abdominal surgery	∆IVC (mm), Caval index (%) MV, Caval index (%) Spont., IVCmax (mm), IVCmin (mm)	∆CO (%)	Crystalloid	56

Abbreviations: ICU, intensive care unit; IVC, inferior vena cava diameter; IJV, internal jugular vein; IVCDV, inferior vena cava diameter variation; Spont, spontaneous; MV, mechanical ventilation; CO, cardiac output; MABP, mean arterial blood pressure; CI, cardiac index; VTI, velocity time integral; SV, stroke volume.

## Data Availability

The original contributions presented in the study are included in the article/Supplementary Materials. Further inquiries can be directed to the corresponding author.

## Supplementary Materials

The following supporting information can be downloaded at: https://www.mdpi.com/article/10.3390/jcm14020492/s1, Supplementary S1: Search strategy for each database; Supplementary S2: PICOS criteria; Table S1: PRISMA-NMA Checklist; Table S2: Risk of bias explanation; Table S3: Major exclusions with reason for exclusion; Table S4: Additional characteristics and outcomes; Table S5: Summary of outcome data in included studies (CINeMA network meta-analysis); Table S6: Network, test parameters and direct comparisons characteristics; Table S7: League table; Table S8: League table for studies with low-moderate bias; Table S9: League table for sensitivity analysis (by caval index); Table S10: Meta-regression; Table S11: The estimated values of between-study variance and incoherence for the network meta-analysis; Table S12: Per-comparison contribution matrix; Table S13: Percentage contribution matrix; Table S14: Risk of bias analysis; Figure S1: SUCRA radial plot and ranking values; Figure S2: Risk of bias bar chart for studied comparisons; Figure S3: Network meta-analysis regression plot; Figure S4: Certainty of evidence assessment (CINeMA approach).
