# Supplementary material for: Assessment of Fluid Responsiveness via Central Venous Ultrasound Measurement: A Network Meta-Analysis
Source: J Clin Med. 2025 Jan 14;14(2):492. doi: 10.3390/jcm14020492 (PMC11765712; doi:10.3390/jcm14020492)
Supplement: Supplementary file 1 [file jcm-14-00492-s001.zip › jcm-3396316-supplementary.pdf]

Supplementary Material

Supplementary S1

Search strategy for each database.

PubMed, MEDLINE: (“fluid responsiveness” OR “fluid resuscitation” OR “volume responsiveness” OR “fluid status” OR “volume status” OR “volemic status”) AND (‘intensive care’ OR ‘critical care’) AND (caval OR “inferior vena cava” OR IVC OR “passive leg raising” OR PLR OR “fluid challenge” OR “fluid bolus” OR “central venous pressure” OR CVP OR “central venous” OR “pulse pressure variation” OR “stroke volume variation”) AND (AUC OR AUROC OR ROC)

Cochrane CENTRAL: ("fluid responsiveness" OR "fluid resuscitation" OR "volume responsiveness" OR "fluid status" OR "volume status" OR "volemic status") AND ("intensive care" OR "critical care") AND (caval OR "inferior vena cava" OR IVC OR "passive leg raising" OR PLR OR "fluid challenge" OR "fluid bolus" OR "central venous pressure" OR CVP OR "central venous" OR "pulse pressure variation" OR "stroke volume variation") AND (AUC OR AUROC OR ROC)

Supplementary S2

PICOS criteria.

The PICOS criteria outline the framework for defining key components of a systematic review or meta-analysis: Population (P) – the target group studied, Intervention (I) – the treatment or exposure assessed, Comparison (C) – the control or alternative intervention, Outcomes (O) – the results measured, and Study design (S) – the type of studies included.

Population: adult patients. **Index test (test method):** ultrasound measures of central veins: inferior vena cava (IVC), superior vena cava, subclavian vena, jugular vena. **Comparator ('gold standard' method):** fluid challenge (FC) method for fluid responsiveness assessment. **Outcomes:** area under the receiver operating characteristic (AUROC) for cut-off value. **Study design:** prospective cohort studies. **Time range:** from inception to March 2024.

Table S1. PRISMA NMA Checklist.

Section/Topic	Item #	Checklist Item	Reported on Page #
**TITLE**			
Title	1	Identify the report as a systematic review *incorporating a network meta-analysis (or related form of meta-analysis).*	Title page
**ABSTRACT**			
Structured summary	2	Provide a structured summary including, as applicable:<br>**Background:** main objectives<br>**Methods:** data sources; study eligibility criteria, participants, and interventions; study appraisal; and *synthesis methods, such as network meta-analysis.*<br>**Results:** number of studies and participants identified; summary estimates with corresponding confidence/credible intervals; *treatment rankings may also be discussed. Authors may choose to summarize pairwise comparisons against a chosen treatment included in their analyses for brevity.*<br>**Discussion/Conclusions:** limitations; conclusions and implications of findings.<br>**Other:** primary source of funding; systematic review registration number with registry name.	Abstract
**INTRODUCTION**			
Rationale	3	Describe the rationale for the review in the context of what is already known*, including mention of why a network meta-analysis has been conducted.*	Introduction
Objectives	4	Provide an explicit statement of questions being addressed, with reference to participants, interventions, comparisons, outcomes, and study design (PICOS).	Introduction
**METHODS**			
Protocol and registration	5	Indicate whether a review protocol exists and if and where it can be accessed (e.g., Web address); and, if available, provide registration information, including registration number.	Abstract, methods
Eligibility criteria	6	Specify study characteristics (e.g., PICOS, length of follow-up) and report characteristics (e.g., years considered, language, publication status) used as criteria for eligibility, giving rationale. *Clearly describe eligible treatments included in the treatment network, and note whether any have been clustered or merged into the same node (with justification).*	Methods, Supplement (PICOS)
Information sources	7	Describe all information sources (e.g., databases with dates of coverage, contact with study authors to identify additional studies) in the search and date last searched.	Methods
Search	8	Present full electronic search strategy for at least one database, including any limits used, such that it could be repeated.	Methods, Supplement
Study selection	9	State the process for selecting studies (i.e., screening, eligibility, included in systematic review, and, if applicable, included in the meta-analysis).	Methods
Data collection process	10	Describe method of data extraction from reports (e.g., piloted forms, independently, in duplicate) and any processes for obtaining and confirming data from investigators.	Methods
Data items	11	List and define all variables for which data were sought (e.g., PICOS, funding sources) and any assumptions and simplifications made.	Methods
Geometry of the network	S1	Describe methods used to explore the geometry of the treatment network under study and potential biases related to it. This should include how the evidence base has been graphically summarized for presentation, and what characteristics were compiled and used to describe the evidence base to readers.	Methods, Fig. 2
Risk of bias within individual studies	12	Describe methods used for assessing risk of bias of individual studies (including specification of whether this was done at the study or outcome level), and how this information is to be used in any data synthesis.	Methods
Summary measures	13	State the principal summary measures (e.g., risk ratio, difference in means). *Also describe the use of additional summary measures assessed, such as treatment rankings and surface under the cumulative ranking curve (SUCRA) values, as well as modified approaches used to present summary findings from meta-analyses.*	Methods
Planned methods of analysis	14	Describe the methods of handling data and combining results of studies for each network meta-analysis. This should include, but not be limited to:<br>● *Handling of multi-arm trials;*<br>● *Selection of variance structure;*<br>● *Selection of prior distributions in Bayesian analyses; and*<br>● *Assessment of model fit.*	Methods
Assessment of Inconsistency	S2	Describe the statistical methods used to evaluate the agreement of direct and indirect evidence in the treatment network(s) studied. Describe efforts taken to address its presence when found.	Methods
Risk of bias across studies	15	Specify any assessment of risk of bias that may affect the cumulative evidence (e.g., publication bias, selective reporting within studies).	Methods

Additional analyses	16	Describe methods of additional analyses if done, indicating which were pre-specified. This may include, but not be limited to, the following:<br>● Sensitivity or subgroup analyses;<br>● Meta-regression analyses;<br>● *Alternative formulations of the treatment network; and*<br>● *Use of alternative prior distributions for Bayesian analyses (if applicable).*	Methods
**RESULTS†**			
Study selection	17	Give numbers of studies screened, assessed for eligibility, and included in the review, with reasons for exclusions at each stage, ideally with a flow diagram.	Results, Fig. 1
Presentation of network structure	S3	Provide a network graph of the included studies to enable visualization of the geometry of the treatment network.	Supplement, Fig. 2
Summary of network geometry	S4	Provide a brief overview of characteristics of the treatment network. This may include commentary on the abundance of trials and randomized patients for the different interventions and pairwise comparisons in the network, gaps of evidence in the treatment network, and potential biases reflected by the network structure.	Supplement, Fig, 2
Study characteristics	18	For each study, present characteristics for which data were extracted (e.g., study size, PICOS, follow-up period) and provide the citations.	Supplement, Table 1
Risk of bias within studies	19	Present data on risk of bias of each study and, if available, any outcome level assessment.	Supplement, Results
Results of individual studies	20	For all outcomes considered (benefits or harms), present, for each study: 1) simple summary data for each intervention group, and 2) effect estimates and confidence intervals. *Modified approaches may be needed to deal with information from larger networks.*	Supplement
Synthesis of results	21	Present results of each meta-analysis done, including confidence/credible intervals. *In larger networks, authors may focus on comparisons versus a particular comparator (e.g. placebo or standard care), with full findings presented in an appendix. League tables and forest plots may be considered to summarize pairwise comparisons.* If additional summary measures were explored (such as treatment rankings), these should also be presented.	Results, Supplement
Exploration for inconsistency	S5	Describe results from investigations of inconsistency. This may include such information as measures of model fit to compare consistency and inconsistency models, *P* values from statistical tests, or summary of inconsistency estimates from different parts of the treatment network.	Supplement, Results
Risk of bias across studies	22	Present results of any assessment of risk of bias across studies for the evidence base being studied.	Supplement, Results
Results of additional analyses	23	Give results of additional analyses, if done (e.g., sensitivity or subgroup analyses, meta-regression analyses*, alternative network geometries studied, alternative choice of prior distributions for Bayesian analyses,* and so forth).	Results
DISCUSSION			
Summary of evidence	24	Summarize the main findings, including the strength of evidence for each main outcome; consider their relevance to key groups (e.g., healthcare providers, users, and policy-makers).	Discussion
Limitations	25	Discuss limitations at study and outcome level (e.g., risk of bias), and at review level (e.g., incomplete retrieval of identified research, reporting bias). *Comment on the validity of the assumptions, such as transitivity and consistency. Comment on any concerns regarding network geometry (e.g., avoidance of certain comparisons).*	Discussion
Conclusions	26	Provide a general interpretation of the results in the context of other evidence, and implications for future research.	Conclusions
FUNDING			
Funding	27	Describe sources of funding for the systematic review and other support (e.g., supply of data); role of funders for the systematic review. This should also include information regarding whether funding has been received from manufacturers of treatments in the network and/or whether some of the authors are content experts with professional conflicts of interest that could affect use of treatments in the network.	Title

Table S2. Risk of bias explanation.

This section provides information on the bias risk assessment framework used in this study

Adapted QUADAS-2 questions

Domain 1: Patient selection
Risk of bias: could the selection of patients have introduced bias?
Was a consecutive or random sample of patients enrolled?
Was a case–control design avoided?
Did the study avoid inappropriate exclusions?
A clear objective and description of inclusion criteria of the study population?
A detailed description of the study population?
Domain 1: Applicability
Are there concerns that the included patients and setting do not match the review question?

Domain 2: Index test (test method)
Risk of Bias: Could the Conduct or Interpretation of the Index Test Have Introduced Bias?
Were the index test results interpreted without knowledge of the results of the reference standard?
Domain 2: Applicability
Are there concerns that the index test, its conduct, or interpretation differ from the review question?

Domain 3: Reference standard (gold standard method)
Risk of Bias: Could the Reference Standard, Its Conduct, or Its Interpretation Have Introduced Bias?
Is the reference standard likely to correctly classify the target condition?
Domain 3: Applicability
Are there concerns that the target condition as defined by the reference standard does not match the question?

Domain 4: Flow and timing
Risk of Bias: Could the Patient Flow Have Introduced Bias?
Was there an appropriate interval between the index test and reference standard?
Did all patients receive the same reference standard?
Were all patients included in the analysis?

We employed an average bias method to calculate the overall risk of bias. For each study, a sum score was computed, considering the following criteria: ☺ was assigned a score of 0 and ☹ a score of 1. We implemented a rule that the overall risk of bias considered ‘Low’ of if sum score = 0, ‘Moderate’ if sum score = 1 and ‘High’ of sum score 2 and more.

Table S3. Major exclusions with reason for exclusion.

Cause of exclusion	Study
**no relevant data for NMA**	1. Lanspa MJ, Grissom CK, Hirshberg EL, Jones JP, Brown SM. Applying dynamic parameters to predict hemodynamic response to volume expansion in spontaneously breathing patients with septic shock. Shock. 2013 Feb;39(2):155-60. doi: 10.1097/SHK.0b013e31827f1c6a. PMID: 23324885; PMCID: PMC3580843.<br>2. Wang Y, Jiang Y, Wu H, Wang R, Wang Y, Du C. Assessment of fluid responsiveness by inferior vena cava diameter variation in post-pneumonectomy patients. Echocardiography. 2018 Dec;35(12):1922-1925. doi: 10.1111/echo.14172. Epub 2018 Oct 18. PMID: 30338549; PMCID: PMC6587495.<br>3. de Oliveira OH, Freitas FG, Ladeira RT, Fischer CH, Bafi AT, Azevedo LC, Machado FR. Comparison between respiratory changes in the inferior vena cava diameter and pulse pressure variation to predict fluid responsiveness in postoperative patients. J Crit Care. 2016 Aug;34:46-9. doi: 10.1016/j.jcrc.2016.03.017. Epub 2016 Mar 30. PMID: 27288609.<br>4. Preau S, Bortolotti P, Colling D, Dewavrin F, Colas V, Voisin B, Onimus T, Drumez E, Durocher A, Redheuil A, Saulnier F. Diagnostic Accuracy of the Inferior Vena Cava Collapsibility to Predict Fluid Responsiveness in Spontaneously Breathing Patients With Sepsis and Acute Circulatory Failure. Crit Care Med. 2017 Mar;45(3):e290-e297. doi: 10.1097/CCM.0000000000002090. PMID: 27749318.<br>5. Mohiedden, Mohammad & Abdelfattah, & Elgammal, Sahar & Elsayed, Khaled & Abdalla, Radwa & Mowafy, Sherif. (2020). Distensibility Index of Inferior Vena Cava and Pulse Pressure Variation as Predictors of Fluid Responsiveness in Mechanically Ventilated Shocked Patients. Journal of Emergency Medicine, Trauma and Acute Care. 2020. 10.5339/jemtac.2020.2.<br>6. Elnakera AM, Abdullah RM, Matar HM. End-tidal carbon dioxide's change to fluid challenge versus internal jugular vein dispensability index for predicting fluid responsiveness in septic patients: A prospective, observational study. Indian J Anaesth. 2023 Jun;67(6):537-543. doi: 10.4103/ija.ija_52_23. Epub 2023 Jun 14. PMID: 37476446; PMCID: PMC10355349.<br>7. He F, Li X, Thapa S, Li C, Luo J, Dai W, Liu J. Evaluation of volume responsiveness by pulse pressure variability and inferior vena cava dispensability index at different tidal volumes by mechanical ventilation. Braz J Med Biol Res. 2019;52(9):e8827. doi: 10.1590/1414-431X20198827. Epub 2019 Aug 29. PMID: 31482978; PMCID: PMC6720221.<br>8. Lu N, Xi X, Jiang L, Yang D, Yin K. Exploring the best predictors of fluid responsiveness in patients with septic shock. Am J Emerg Med. 2017 Sep;35(9):1258-1261. doi: 10.1016/j.ajem.2017.03.052. Epub 2017 Mar 22. PMID: 28363617.<br>9. Corl KA, George NR, Romanoff J, Levinson AT, Chheng DB, Merchant RC, Levy MM, Napoli AM. Inferior vena cava collapsibility detects fluid responsiveness among spontaneously breathing critically-ill patients. J Crit Care. 2017 Oct;41:130-137. doi: 10.1016/j.jcrc.2017.05.008. Epub 2017 May 12. PMID: 28525778.<br>10. Nagi, A.I., Shafik, A.M., Fatah, A.M.A. et al. Inferior vena cava collapsibility index as a predictor of fluid responsiveness in sepsis-related acute circulatory failure. Ain-Shams J Anesthesiol 13, 75 (2021). https://doi.org/10.1186/s42077-021-00194-y<br>11. Zhao J, Wang G. Inferior Vena Cava Collapsibility Index is a Valuable and Non-Invasive Index for Elevated General Heart End-Diastolic Volume Index Estimation in Septic Shock Patients. Med Sci Monit. 2016 Oct 20;22:3843-3848. doi: 10.12659/msm.897406. PMID: 27762259; PMCID: PMC5085335.<br>12. Theerawit P, Morasert T, Sutherasan Y. Inferior vena cava diameter variation compared with pulse pressure variation as predictors of fluid responsiveness in patients with sepsis. J Crit Care. 2016 Dec;36:246-251. doi: 10.1016/j.jcrc.2016.07.023. Epub 2016 Aug 13. PMID: 27591389.<br>13. Moretti R, Pizzi B. Inferior vena cava distensibility as a predictor of fluid responsiveness in patients with subarachnoid hemorrhage. Neurocrit Care. 2010 Aug;13(1):3-9. doi: 10.1007/s12028-010-9356-z. PMID: 20373051.<br>14. Machare-Delgado E, Decaro M, Marik PE. Inferior vena cava variation compared to pulse contour analysis as predictors of fluid responsiveness: a prospective cohort study. J Intensive Care Med. 2011 Mar-Apr;26(2):116-24. doi: 10.1177/0885066610384192. PMID: 21595098.<br>15. Guarracino F, Ferro B, Forfori F, Bertini P, Magliacano L, Pinsky MR. Jugular vein distensibility predicts fluid responsiveness in septic patients. Crit Care. 2014 Dec 5;18(6):647. doi: 10.1186/s13054-014-0647-1. PMID: 25475099; PMCID: PMC4301660.<br>16. Corl KA, Azab N, Nayeemuddin M, Schick A, Lopardo T, Zeba F, Phillips G, Baird G, Merchant RC, Levy MM, Blaivas M, Abbasi A. Performance of a 25% Inferior Vena Cava Collapsibility in Detecting Fluid Responsiveness When Assessed by Novice Versus Expert Physician Sonologists. J Intensive Care Med. 2020 Dec;35(12):1520-1528. doi: 10.1177/0885066619881123. Epub 2019 Oct 14. PMID: 31610729; PMCID: PMC7153972.<br>17. Sawe HR, Haeffele C, Mfinanga JA, Mwafongo VG, Reynolds TA. Predicting Fluid Responsiveness Using Bedside Ultrasound Measurements of the Inferior Vena Cava and Physician Gestalt in the Emergency Department of an Urban Public Hospital in Sub-Saharan Africa. PLoS One. 2016 Sep 27;11(9):e0162772. doi: 10.1371/journal.pone.0162772. PMID: 27677085; PMCID: PMC5038941.<br>18. Charbonneau H, Riu B, Faron M, Mari A, Kurrek MM, Ruiz J, Geeraerts T, Fourcade O, Genestal M, Silva S. Predicting preload responsiveness using simultaneous recordings of inferior and superior vena cavae diameters. Crit Care. 2014 Sep 5;18(5):473. doi: 10.1186/s13054-014-0473-5. PMID: 25189403; PMCID: PMC4175634.

	19. Feissel M, Michard F, Mangin I, Ruyer O, Faller JP, Teboul JL. Respiratory changes in aortic blood velocity as an indicator of fluid responsiveness in ventilated patients with septic shock. Chest. 2001 Mar;119(3):867-73. doi: 10.1378/chest.119.3.867. PMID: 11243970.<br>20. Barbier C, Loubières Y, Schmit C, Hayon J, Ricôme JL, Jardin F, Vieillard-Baron A. Respiratory changes in inferior vena cava diameter are helpful in predicting fluid responsiveness in ventilated septic patients. Intensive Care Med. 2004 Sep;30(9):1740-6. doi: 10.1007/s00134-004-2259-8. Epub 2004 Mar 18. PMID: 15034650.<br>21. Giraud R, Abraham PS, Brindel P, Siegenthaler N, Bendjelid K. Respiratory changes in subclavian vein diameters predicts fluid responsiveness in intensive care patients: a pilot study. J Clin Monit Comput. 2018 Dec;32(6):1049-1055. doi: 10.1007/s10877-018-0103-x. Epub 2018 Jan 29. PMID: 29380189.<br>22. Ibarra-Estrada MÁ, López-Pulgarín JA, Mijangos-Méndez JC, Díaz-Gómez JL, Aguirre-Avalos G. Respiratory variation in carotid peak systolic velocity predicts volume responsiveness in mechanically ventilated patients with septic shock: a prospective cohort study. Crit Ultrasound J. 2015 Dec;7(1):29. doi: 10.1186/s13089-015-0029-1. Epub 2015 Jun 26. PMID: 26123610; PMCID: PMC4485670.<br>23. Muller L, Bobbia X, Toumi M, Louart G, Molinari N, Ragonnet B, Quintard H, Leone M, Zoric L, Lefrant JY; AzuRea group. Respiratory variations of inferior vena cava diameter to predict fluid responsiveness in spontaneously breathing patients with acute circulatory failure: need for a cautious use. Crit Care. 2012 Oct 8;16(5):R188. doi: 10.1186/cc11672. PMID: 23043910; PMCID: PMC3682290.<br>24. Kumar A, Mahendran M, Hari S, Ranjan P, Soneja M, Wig N. Subclavian vein collapsibility as a predictor of fluid responsiveness in spontaneously breathing hypotensive patients. J Assoc Physicians India. 2022 Apr;70(4):11-12. PMID: 35443482.<br>25. de Valk S, Olgers TJ, Holman M, Ismael F, Ligtenberg JJ, Ter Maaten JC. The caval index: an adequate non-invasive ultrasound parameter to predict fluid responsiveness in the emergency department? BMC Anesthesiol. 2014 Dec 12;14:114. doi: 10.1186/1471-2253-14-114. PMID: 25844064; PMCID: PMC4384238.<br>26. Feissel M, Michard F, Faller JP, Teboul JL. The respiratory variation in inferior vena cava diameter as a guide to fluid therapy. Intensive Care Med. 2004 Sep;30(9):1834-7. doi: 10.1007/s00134-004-2233-5. Epub 2004 Mar 25. PMID: 15045170.<br>27. Bubenek-Turconi ŞI, Hendy A, Băilă S, Drăgan A, Chioncel O, Văleanu L, Moroșanu B, Iliescu VA. The value of a superior vena cava collapsibility index measured with a miniaturized transoesophageal monoplane continuous echocardiography probe to predict fluid responsiveness compared to stroke volume variations in open major vascular surgery: a prospective cohort study. J Clin Monit Comput. 2020 Jun;34(3):491-499. doi: 10.1007/s10877-019-00346-4. Epub 2019 Jul 5. PMID: 31278544; PMCID: PMC7223808.<br>28. Sobczyk D, Nycz K, Andruszkiewicz P, Wierzbicki K, Stapor M. Ultrasonographic caval indices do not significantly contribute to predicting fluid responsiveness immediately after coronary artery bypass grafting when compared to passive leg raising. Cardiovasc Ultrasound. 2016 Jun 8;14(1):23. doi: 10.1186/s12947-016-0065-4. PMID: 27267175; PMCID: PMC4897915.<br>29. Zhang X, Feng J, Zhu P, Luan H, Wu Y, Zhao Z. Ultrasonographic measurements of the inferior vena cava variation as a predictor of fluid responsiveness in patients undergoing anesthesia for surgery. J Surg Res. 2016 Jul;204(1):118-22. doi: 10.1016/j.jss.2016.03.036. Epub 2016 Mar 24. PMID: 27451877.<br>30. Murthi SB, Fatima S, Menne AR, Glaser JJ, Galvagno SM, Biederman S, Fang R, Chen H, Scalea TM. Ultrasound assessment of volume responsiveness in critically ill surgical patients: Two measurements are better than one. J Trauma Acute Care Surg. 2017 Mar;82(3):505-511. doi: 10.1097/TA.0000000000001331. PMID: 28030505.
**no relevant gold standard**	1. Zhang H, Liu D, Wang X, Zhang Q, Tang B, Ding X, Chen H, Wu J. [Does Inferior Vena Cava variability predict fluid responsiveness in critically ill patients with atrial fibrillation]. Zhonghua Yi Xue Za Zhi. 2015 May 19;95(19):1453-6. Chinese. PMID: 26178491.<br>2. Pişkin Ö, Öz İİ. Accuracy of pleth variability index compared with inferior vena cava diameter to predict fluid responsiveness in mechanically ventilated patients. Medicine (Baltimore). 2017 Nov;96(47):e8889. doi: 10.1097/MD.0000000000008889. PMID: 29382017; PMCID: PMC5709016.<br>3. Baloch K, Rehman Memon A, Ikhlaq U, Umair M, Ansari MI, Abubaker J, Salahuddin N. Assessing the Utility of End-Tidal Carbon Dioxide as a Marker for Fluid Responsiveness in Cardiogenic Shock. Cureus. 2021 Feb 5;13(2):e13164. doi: 10.7759/cureus.13164. PMID: 33692926; PMCID: PMC7938016.<br>4. Corl K, Napoli AM, Gardiner F. Bedside sonographic measurement of the inferior vena cava caval index is a poor predictor of fluid responsiveness in emergency department patients. Emerg Med Australas. 2012 Oct;24(5):534-9. doi: 10.1111/j.1742-6723.2012.01596.x. Epub 2012 Sep 7. PMID: 23039295.<br>5. Iizuka Y, Nomura T, Sanui M, Mochida Y, Aomatsu A, Lefor AK. Collapsibility of the Right Internal Jugular Vein Predicts Responsiveness to Fluid Administration in Patients Receiving Pressure Support Ventilation: A Prospective Cohort Study. J Clin Med Res. 2020 Mar;12(3):150-156. doi: 10.14740/jocmr4064. Epub 2020 Mar 2. PMID: 32231750; PMCID: PMC7092757.<br>6. Pereira RM, Silva AJLCD, Faller J, Gomes BC, Silva JM Jr. Comparative Analysis of the Collapsibility Index and Distensibility Index of the Inferior Vena Cava Through Echocardiography with Pulse Pressure Variation That Predicts Fluid Responsiveness in Surgical Patients: An Observational Controlled Trial. J Cardiothorac Vasc Anesth. 2020 Aug;34(8):2162-2168. doi: 10.1053/j.jvca.2020.02.007. Epub 2020 Feb 12. PMID: 32217045.<br>7. Vignon P, Repessé X, Bégot E, Léger J, Jacob C, Bouferrache K, Slama M, Prat G, Vieillard-Baron A. Comparison of Echocardiographic Indices Used to Predict Fluid Responsiveness in Ventilated Patients. Am J Respir Crit Care Med. 2017 Apr 15;195(8):1022-1032. doi: 10.1164/rccm.201604-0844OC. PMID: 27653798.<br>8. Horejsek J, Balík M, Kunstýř J, Michálek P, Kopecký P, Brožek T, Bartošová T, Fink A, Waldauf P, Porizka M. Internal jugular vein collapsibility does not predict fluid responsiveness in spontaneously breathing patients after cardiac surgery. J Clin Monit Comput. 2023 Dec;37(6):1563-1571. doi: 10.1007/s10877-023-01066-6. Epub 2023 Aug 12. PMID: 37572237.<br>9. Nedel WL, Simas DM, Marin LG, Morais VD, Friedman G. Respiratory Variation in Femoral Vein Diameter Has Moderate Accuracy as a Marker of Fluid Responsivity in Mechanically Ventilated Septic Shock Patients.

	Ultrasound Med Biol. 2017 Nov;43(11):2713-2717. doi: 10.1016/j.ultrasmedbio.2017.06.023. Epub 2017 Jul 27. PMID: 28756901.<br>10. Zhang H, Zhang Q, Chen X, Wang X, Liu D; Chinese Critical Ultrasound Study Group (CCUSG). Respiratory variations of inferior vena cava fail to predict fluid responsiveness in mechanically ventilated patients with isolated left ventricular dysfunction. Ann Intensive Care. 2019 Oct 7;9(1):113. doi: 10.1186/s13613-019-0589-5. PMID: 31591663; PMCID: PMC6779682.<br>11. Broilo F, Meregalli A, Friedman G. Right internal jugular vein distensibility appears to be a surrogate marker for inferior vena cava vein distensibility for evaluating fluid responsiveness. Rev Bras Ter Intensiva. 2015 Jul-Sep;27(3):205-11. doi: 10.5935/0103-507X.20150042. PMID: 26465243; PMCID: PMC4592113.<br>12. Haliloğlu M, Bilgili B, Kararmaz A, Cinel İ. The value of internal jugular vein collapsibility index in sepsis. Ulus Travma Acil Cerrahi Derg. 2017 Jul;23(4):294-300. doi: 10.5505/tjtes.2016.04832. PMID: 28762449.<br>13. Yao B, Liu JY, Sun YB, Zhao YX, Li LD. The Value of the Inferior Vena Cava Area Distensibility Index and its Diameter Ratio for Predicting Fluid Responsiveness in Mechanically Ventilated Patients. Shock. 2019 Jul;52(1):37-42. doi: 10.1097/SHK.0000000000001238. PMID: 31188800.<br>14. Doucet JJ, Ferrada P, Murthi S, Nirula R, Edwards S, Cantrell E, Han J, Haase D, Singleton A, Birkas Y, Casola G, Coimbra R; AAST Multi-Institutional Trials Committee. Ultrasonographic inferior vena cava diameter response to trauma resuscitation after 1 hour predicts 24-hour fluid requirement. J Trauma Acute Care Surg. 2020 Jan;88(1):70-79. doi: 10.1097/TA.0000000000002525. PMID: 31688824.
**not connected network (atypical test parameter)**	1. Vieillard-Baron A, Chergui K, Rabiller A, Peyrouset O, Page B, Beauchet A, Jardin F. Superior vena caval collapsibility as a gauge of volume status in ventilated septic patients. Intensive Care Med. 2004 Sep;30(9):1734-9. doi: 10.1007/s00134-004-2361-y. Epub 2004 Jun 26. PMID: 15375649.

Table S4. Additional characteristics and outcomes.

This table provides additional information extracted from the publications included in this systematic review and meta-analysis

Study	Sample size	Test parameter	Test criterion (cut-off)	Gold standard additional characteristics	Gold standard parameter	Gold standard criterion	Prevalence (% of R)	R	NR	Mean age	Men, %	BMI, kg/m2	CVP, mean, mmHg	AUROC	AUROC lower limit	AUROC upper limit	AUROC SD
Airapetian N., 2015 [33]	59	Caval index (%) Spont	42	500 ml	ΔCO (%)	10	49	29	30	57	51	ND	ND	0.62	0.49	0.74	0.07
Airapetian N., 2015 [33]	59	IVCmax (mm)	21	500 ml	ΔCO (%)	10	49	29	30	57	51	ND	ND	0.62	0.49	0.75	0.07
Ismail M., 2022 [34]	102	Caval index (%) Spont	40	500 ml	MABP (mmHg)	70	43	44	58	62.9	57.8	ND	ND	0.908	0.84	0.975	0.34
Ismail M., 2022 [34]	102	IVCmax (mm)	13	500 ml	MABP (mmHg)	70	43	44	58	62.9	57.8	ND	ND	0.742	0.642	0.841	0.51
Ismail M., 2022 [34]	102	IVCmin (mm)	5	500 ml	MABP (mmHg)	70	43	44	58	62.9	57.8	ND	ND	0.857	0.773	0.94	0.43
Doucet J.J., 2020 [35]	115	Caval index (%) Spont	ND	R: 433±519, NR: 410±466	IVCD (mm)	10	ND	ND	ND	ND	ND	ND	ND	0.75	0.66	0.85	0.27
Doucet J.J., 2020 [35]	77	IJV index (%)	ND	R: 433±519, NR: 410±466	IVCD (mm)	10	ND	ND	ND	ND	ND	ND	ND	0.54	0.42	0.67	0.29
Doucet J.J., 2020 [35]	78	IJVmax (mm)	ND	R: 433±519, NR: 410±466	IVCD (mm)	10	ND	ND	ND	ND	ND	ND	ND	0.46	ND	ND	0.35
Doucet J.J., 2020 [35]	103	IJVmin (mm)	ND	R: 433±519, NR: 410±466	IVCD (mm)	10	ND	ND	ND	ND	ND	ND	ND	0.48	0.24	0.6	0.36
Doucet J.J., 2020 [35]	123	IVCmax (mm)	ND	R: 433±519, NR: 410±466	IVCD (mm)	10	ND	ND	ND	ND	ND	ND	ND	0.69	ND	ND	0.34
Elsaeed A., 2022 [36]	40	Caval index (%) Spont	35	7 ml/kg	ΔCI (%)	15	60	24	16	58.45	52.5	ND	ND	0.97	0.861	0.999	0.22
Elsaeed A., 2022 [36]	40	IJV index (%)	17.56	7 ml/kg	ΔCI (%)	15	60	24	16	58.45	52.5	ND	ND	0.969	0.859	0.999	0.22
Bortolotti P., 2018 [37]	55	Caval index (%) Spont	39	500 ml	ΔVTI (%)	10	53	29	26	68.36	63.6	25.05	ND	0.93	0.86	1	0.26
Bortolotti P., 2018 [37]	55	IVCmax (mm)	18	500 ml	ΔVTI (%)	10	53	29	26	68.36	63.6	25.05	ND	0.83	0.72	0.94	0.41
Bortolotti P., 2018 [37]	55	IVCmin (mm)	11	500 ml	ΔVTI (%)	10	53	29	26	68.36	63.6	25.05	ND	0.93	0.86	1	0.26
Taccheri T., 2021 [38]	30	Caval index (%) MV (Vt=6 mL/kg)	ND	500 ml	ΔCI (%)	10	50	15	15	66.5	76.7	ND	9.5	0.64	0.6	0.679	0.11
Taccheri T., 2021 [38]	30	IVCmax (mm)	ND	500 ml	ΔCI (%)	10	50	15	15	66.5	76.7	ND	9.5	0.58	ND	ND	0.11
Taccheri T., 2021 [38]	30	ΔCaval index (%)	4	500 ml	ΔCI (%)	10	50	15	15	66.5	76.7	ND	9.5	0.88	ND	ND	0.06
Taccheri T., 2021 [38]	30	ΔIVCDV (mm)	ND	500 ml	ΔCI (%)	10	50	15	15	66.5	76.7	ND	9.5	0.56	ND	ND	0.11
Ma G.G., 2018 [39]	70	Caval index (%) MV (Vt=8 mL/kg; PEEP = 5 cmH2O)	13.39	500 ml	ΔSV (%)	15	50	35	35	61	62.9	22	11.5	0.83	0.72	0.91	0.4
Ma G.G., 2018 [39]	70	IJV index (%)	12.99	500 ml	ΔSV (%)	15	50	35	35	61	62.9	22	11.5	0.88	0.78	0.94	0.34
Ma G.G., 2018 [39]	70	IJVmax (mm)	8.6	500 ml	ΔSV (%)	15	50	35	35	61	62.9	22	11.5	0.55	0.43	0.67	0.5
Ma G.G., 2018 [39]	70	IJVmin (mm)	6.4	500 ml	ΔSV (%)	15	50	35	35	61	62.9	22	11.5	0.55	0.43	0.67	0.5
Ma G.G., 2018 [39]	70	IVCmax (mm)	15.7	500 ml	ΔSV (%)	15	50	35	35	61	62.9	22	11.5	0.53	0.4	0.65	0.52
Ma G.G., 2018 [39]	70	IVCmin (mm)	14	500 ml	ΔSV (%)	15	50	35	35	61	62.9	22	11.5	0.58	0.46	0.7	0.5
Baker A.K., 2013 [40]	25	Caval index (%) MV (Vt<8 mL/kg)	ND	500 ml	ΔSV (%)	15	48	12	13	60.2	68	ND	10.07	0.46	0.22	0.69	0.28
Baker A.K., 2013 [40]	25	IVCmin (mm)	ND	500 ml	ΔSV (%)	15	48	12	13	60.2	68	ND	10.07	0.55	0.31	0.79	0.29
Ma Q., 2022 [41]	56	ΔIVC (mm)	2.9	5ml/kg	ΔCO (%)	15	59	33	23	56.82	55.4	ND	4.58	0.8	0.67	0.9	0.43

Ma Q., 2022 [41]	56	Caval index (%) MV (Vt=8 mL/kg; PEEP = 5 cmH2O)	15.32	5ml/kg	ΔCO (%)	15	59	33	23	56.82	55.4	ND	4.58	0.8	0.68	0.9	0.41
Ma Q., 2022 [41]	56	Caval index (%) Spont	30.25	5ml/kg	ΔCO (%)	15	39	22	34	57.39	55.4	ND	2.89	0.87	0.75	0.94	0.35
Ma Q., 2022 [41]	56	IVCmax (mm)	15.3	5ml/kg	ΔCO (%)	15	39	22	34	57.39	55.4	ND	2.89	0.77	0.64	0.87	0.43
Ma Q., 2022 [41]	56	IVCmin (mm)	11.4	5ml/kg	ΔCO (%)	15	39	22	34	57.39	55.4	ND	2.89	0.85	0.73	0.93	0.37

Abbreviations: AUROC, area under the receiver operating characteristic curve; SD, standard deviation; Spont, spontaneous; R, responders; NR, non-responders; ND, no data; ΔCO, change in cardiac output; MABP, mean arterial blood pressure; IVCD, inferior vena cava diameter; ΔCI, change in cardiac index; ΔVTI, change in velocity time integral; ΔSV, change in stroke volume; IVCmax, maximum inferior vena cava diameter; IVCmin, minimum inferior vena cava diameter; IJVmax, maximum internal jugular vein diameter; IJVmin, minimum internal jugular vein diameter; ΔIVCDV, change in inferior vena cava diameter variation; BMI, body mass index; CVP, central venous pressure; MV, mechanical ventilation.

Table S5. Summary of outcome data in included studies (CINeMA network meta-analysis).

This table shows the data that has been entered into the CINeMA web application as a .csv file

Study	ID	Test parameter	AUROC	SD	N	RoB	Indirectness
Airapetian N., 2015 [1]	1	Caval index (%)	0.62	0.07	59	L	1
Airapetian N., 2015 [1]	1	IVCmax (mm)	0.62	0.07	59	L	1
Ismail M., 2022 [2]	2	Caval index (%)	0.908	0.34	102	M	1
Ismail M., 2022 [2]	2	IVCmax (mm)	0.742	0.51	102	M	1
Ismail M., 2022 [2]	2	IVCmin (mm)	0.857	0.43	102	M	1
Doucet J.J., 2020 [3]	3	Caval index (%)	0.75	0.27	115	H	1
Doucet J.J., 2020 [3]	3	IJV index (%)	0.54	0.29	77	H	1
Doucet J.J., 2020 [3]	3	IJVmax (mm)	0.46	0.35	78	H	1
Doucet J.J., 2020 [3]	3	IJVmin (mm)	0.48	0.36	103	H	1
Doucet J.J., 2020 [3]	3	IVCmax (mm)	0.69	0.34	123	H	1
Elsaeed A., 2022 [4]	4	Caval index (%)	0.97	0.22	40	L	1
Elsaeed A., 2022 [4]	4	IJV index (%)	0.969	0.22	40	L	1
Bortolotti P., 2018 [5]	5	Caval index (%)	0.93	0.26	55	L	1
Bortolotti P., 2018 [5]	5	IVCmax (mm)	0.83	0.41	55	L	1
Bortolotti P., 2018 [5]	5	IVCmin (mm)	0.93	0.26	55	L	1
Taccheri T., 2021 [6]	6	Caval index (%)	0.64	0.11	30	M	1
Taccheri T., 2021 [6]	6	IVCmax (mm)	0.58	0.11	30	M	1
Taccheri T., 2021 [6]	6	ΔCaval index (%)	0.88	0.06	30	M	1
Taccheri T., 2021 [6]	6	ΔIVCDV (mm)	0.56	0.11	30	M	1
Ma G.G., 2018 [7]	7	Caval index (%)	0.83	0.4	70	L	1
Ma G.G., 2018 [7]	7	IJV index (%)	0.88	0.34	70	L	1
Ma G.G., 2018 [7]	7	IJVmax (mm)	0.55	0.5	70	L	1
Ma G.G., 2018 [7]	7	IJVmin (mm)	0.55	0.5	70	L	1
Ma G.G., 2018 [7]	7	IVCmax (mm)	0.53	0.52	70	L	1
Ma G.G., 2018 [7]	7	IVCmin (mm)	0.58	0.5	70	L	1
Baker A.K., 2013 [8]	8	Caval index (%)	0.46	0.28	25	M	1
Baker A.K., 2013 [8]	8	IVCmin (mm)	0.55	0.29	25	M	1
Ma Q., 2022 [9]	9	ΔIVC (mm)	0.8	0.43	56	L	1
Ma Q., 2022 [9]	9	Caval index (%)	0.87	0.35	56	L	1
Ma Q., 2022 [9]	9	IVCmax (mm)	0.77	0.43	56	L	1
Ma Q., 2022 [9]	9	IVCmin (mm)	0.85	0.37	56	L	1

Abbreviations: AUROC, area under the receiver operating characteristic curve; SD, standard deviation; IVC, inferior vena cava diameter; IJV, internal jugular vein; IVCDV, inferior vena cava diameter variation; RoB, Risk of Bias (1-low, 2-moderate, 3-high).

Table S6. Network, test parameters and direct comparisons characteristics.

This table summarises additional characteristics of nodes and comparisons in network meta-analysis

**Network characteristics**	
Number of test parameters	9
Number of Studies	9
Total Number of Patients in Network	1979
Total Possible Pairwise Comparisons	36
Total Number of Pairwise Comparisons with Direct Data	23
Is the network connected?	TRUE
Number of Two-arm Studies	3
Number of multi-Arms Studies	6
Average Outcome	0.7156

Test parameter characteristics

**Prediction method**	**Total no. of studies**	**Total no. of patients**	**Min outcome value**	**Max outcome value**	**Average outcome value**
ΔIVC (mm)	1	56	0.80	0.80	0.80
Caval index (%)	9	552	0.46	0.97	0.80
IJV index (%)	3	187	0.54	0.97	0.76
IJVmax (mm)	2	148	0.46	0.55	0.50
IJVmin (mm)	2	173	0.48	0.55	0.51
IVCmax (mm)	7	495	0.53	0.83	0.69
IVCmin (mm)	5	308	0.55	0.93	0.78
ΔCaval index (%)	1	30	0.88	0.88	0.88
ΔIVCDV (mm)	1	30	0.56	0.56	0.56

Direct comparisons characteristics

**Comparison**	**Total no. of studies**	**Total no. of patients**
ΔIVC (mm) vs. Caval index (%)	1	112
ΔIVC (mm) vs. IVCmax (mm)	1	112
ΔIVC (mm) vs. IVCmin (mm)	1	112
Caval index (%) vs. IJV index (%)	3	412
Caval index (%) vs. IJVmax (mm)	2	333
Caval index (%) vs. IJVmin (mm)	2	358
Caval index (%) vs. IVCmax (mm)	7	982
Caval index (%) vs. IVCmin (mm)	5	616
Caval index (%) vs. ΔCaval index (%)	1	60
Caval index (%) vs. ΔIVCDV (mm)	1	60
IJV index (%) vs. IJVmax (mm)	2	295
IJV index (%) vs. IJVmin (mm)	2	320
IJV index (%) vs. IVCmax (mm)	2	340
IJV index (%) vs. IVCmin (mm)	1	140
IJVmax (mm) vs. IJVmin (mm)	2	321
IJVmax (mm) vs. IVCmax (mm)	2	341
IJVmax (mm) vs. IVCmin (mm)	1	140
IJVmin (mm) vs. IVCmax (mm)	2	366
IJVmin (mm) vs. IVCmin (mm)	1	140
IVCmax (mm) vs. IVCmin (mm)	4	566
IVCmax (mm) vs. ΔCaval index (%)	1	60
IVCmax (mm) vs. ΔIVCDV (mm)	1	60
ΔCaval index (%) vs. ΔIVCDV (mm)	1	60

Abbreviations: IVCD, inferior vena cava diameter; IVC, inferior vena cava; IJV, internal jugular vein diameter; IVCDV, inferior vena cava diameter variation.

Table S7. League table.

This table presents all results of the network meta-analysis as Mean difference (95% CI)

**Caval index (%)**	0.021 (-0.089,0.130)	**0.226 (0.090,0.361)**	**0.214 (0.080,0.348)**	**0.090 (0.013,0.166)**	0.039 (-0.054,0.132)	0.073 (-0.126,0.273)	**-0.225 (-0.390, -0.061)**	0.095 (-0.073,0.263)
-0.021 (-0.130,0.089)	**IJV index (%)**	**0.205 (0.058,0.352)**	**0.194 (0.048,0.339)**	0.069 (-0.050,0.187)	0.018 (-0.114,0.150)	0.053 (-0.170,0.275)	**-0.246 (-0.439, -0.053)**	0.074 (-0.122,0.270)
**-0.226 (-0.361, -0.090)**	**-0.205 (-0.352, -0.058)**	**IJVmax (mm)**	-0.011 (-0.170,0.147)	-0.136 (-0.275,0.003)	**-0.187 (-0.339, -0.035)**	-0.152 (-0.388,0.083)	**-0.451 (-0.658, -0.243)**	-0.131 (-0.341,0.079)
**-0.214 (-0.348, -0.080)**	**-0.194 (-0.339, -0.048)**	0.011 (-0.147,0.170)	**IJVmin (mm)**	-0.125 (-0.262,0.013)	**-0.175 (-0.326, -0.025)**	-0.141 (-0.375,0.093)	**-0.439 (-0.646, -0.233)**	-0.119 (-0.329,0.090)
**-0.090 (-0.166, -0.013)**	-0.069 (-0.187,0.050)	0.136 (-0.003,0.275)	0.125 (-0.013,0.262)	**IVCmax (mm)**	-0.051 (-0.151,0.050)	-0.016 (-0.218,0.185)	**-0.315 (-0.479, -0.150)**	0.005 (-0.163,0.173)
-0.039 (-0.132,0.054)	-0.018 (-0.150,0.114)	**0.187 (0.035,0.339)**	**0.175 (0.025,0.326)**	0.051 (-0.050,0.151)	**IVCmin (mm)**	0.034 (-0.168,0.237)	**-0.264 (-0.447, -0.081)**	0.056 (-0.130,0.242)
-0.073 (-0.273,0.126)	-0.053 (-0.275,0.170)	0.152 (-0.083,0.388)	0.141 (-0.093,0.375)	0.016 (-0.185,0.218)	-0.034 (-0.237,0.168)	**ΔIVC (mm)**	**-0.298 (-0.552, -0.045)**	0.022 (-0.234,0.277)
**0.225 (0.061,0.390)**	**0.246 (0.053,0.439)**	**0.451 (0.243,0.658)**	**0.439 (0.233,0.646)**	**0.315 (0.150,0.479)**	**0.264 (0.081,0.447)**	**0.298 (0.045,0.552)**	**ΔCaval index (%)**	**0.320 (0.134,0.506)**
-0.095 (-0.263,0.073)	-0.074 (-0.270,0.122)	0.131 (-0.079,0.341)	0.119 (-0.090,0.329)	-0.005 (-0.173,0.163)	-0.056 (-0.242,0.130)	-0.022 (-0.277,0.234)	**-0.320 (-0.506, -0.134)**	**ΔIVCDV (mm)**

Abbreviations: IVCD, inferior vena cava diameter; IVC, inferior vena cava; IJV, internal jugular vein diameter; IVCDV, inferior vena cava diameter variation.

Table S8. League table for studies with low-moderate bias.

This table presents all results of the network meta-analysis as Mean difference (95% CI) only for low-moderate bias studies

**Caval index (%)**	-0.098<br>(-0.220, 0.024)	0.177<br>(-0.005, 0.360)	0.177<br>(-0.005, 0.360)	**0.103<br>(0.029, 0.177)**	0.033<br>(-0.051, 0.118)	0.075<br>(-0.107, 0.258)	**-0.218<br>(-0.360, -0.077)**	0.102<br>(-0.044, 0.247)
0.098<br>(-0.024, 0.220)	**IJV index (%)**	**0.275<br>(0.080, 0.470)**	**0.275<br>(0.080, 0.470)**	**0.201<br>(0.067, 0.335)**	0.131<br>(-0.006, 0.269)	0.173<br>(-0.042, 0.389)	-0.120<br>(-0.304, 0.063)	**0.200<br>(0.013, 0.386)**
-0.177<br>(-0.360, 0.005)	**-0.275<br>(-0.470, -0.080)**	**IJVmax (mm)**	0.000<br>(-0.225, 0.225)	-0.074<br>(-0.262, 0.113)	-0.144<br>(-0.333, 0.045)	-0.102<br>(-0.355, 0.151)	**-0.396<br>(-0.623, -0.169)**	-0.076<br>(-0.305, 0.154)
-0.177<br>(-0.360, 0.005)	**-0.275<br>(-0.470, -0.080)**	0.000<br>(-0.225, 0.225)	**IJVmin (mm)**	-0.074<br>(-0.262, 0.113)	-0.144<br>(-0.333, 0.045)	-0.102<br>(-0.355, 0.151)	**-0.396<br>(-0.623, -0.169)**	-0.076<br>(-0.305, 0.154)
**-0.103<br>(-0.177, -0.029)**	**-0.201<br>(-0.335, -0.067)**	0.074<br>(-0.113, 0.262)	0.074<br>(-0.113, 0.262)	**IVCmax (mm)**	-0.070<br>(-0.163, 0.024)	-0.028<br>(-0.212, 0.157)	**-0.322<br>(-0.463, -0.180)**	-0.002<br>(-0.147, 0.144)
-0.033<br>(-0.118, 0.051)	-0.131<br>(-0.269, 0.006)	0.144<br>(-0.045, 0.333)	0.144<br>(-0.045, 0.333)	0.070<br>(-0.024, 0.163)	**IVCmin (mm)**	0.042<br>(-0.143, 0.228)	**-0.252<br>(-0.411, -0.093)**	0.068<br>(-0.094, 0.230)
-0.075<br>(-0.258, 0.107)	-0.173<br>(-0.389, 0.042)	0.102<br>(-0.151, 0.355)	0.102<br>(-0.151, 0.355)	0.028<br>(-0.157, 0.212)	-0.042<br>(-0.228, 0.143)	**ΔIVC (mm)**	**-0.294<br>(-0.520, -0.068)**	0.026<br>(-0.202, 0.254)
**0.218<br>(0.077, 0.360)**	0.120<br>(-0.063, 0.304)	**0.396<br>(0.169, 0.623)**	**0.396<br>(0.169, 0.623)**	**0.322<br>(0.180, 0.463)**	**0.252<br>(0.093, 0.411)**	**0.294<br>(0.068, 0.520)**	**ΔCaval index (%)**	**0.320<br>(0.161, 0.479)**
-0.102<br>(-0.247, 0.044)	**-0.200<br>(-0.386, -0.013)**	0.076<br>(-0.154, 0.305)	0.076<br>(-0.154, 0.305)	0.002<br>(-0.144, 0.147)	-0.068<br>(-0.230, 0.094)	-0.026<br>(-0.254, 0.202)	**-0.320<br>(-0.479, -0.161)**	**ΔIVCDV (mm)**

Abbreviations: IVCD, inferior vena cava diameter; IVC, inferior vena cava; IJV, internal jugular vein diameter; IVCDV, inferior vena cava diameter variation.

Table S9. League table for sensitivity analysis (by caval index).

This table presents all results of the network meta-analysis as Mean difference (95% CI) taking into account Caval index estimation in patient on MV or with spontaneous breathing

**Caval index (%) MV**	-0.027 (-0.155, 0.101)	-0.003 (-0.152, 0.147)	**0.204 (0.037, 0.371)**	**0.193 (0.026, 0.359)**	0.069 (-0.047, 0.185)	0.016 (-0.108, 0.140)	0.038 (-0.178, 0.254)	**-0.236 (-0.421, -0.050)**	0.084 (-0.104, 0.273)
0.027 (-0.101, 0.155)	**Caval Index (%) Spont**	0.024 (-0.100, 0.148)	**0.231 (0.079, 0.383)**	**0.220 (0.069, 0.370)**	**0.096 (0.002, 0.190)**	0.043 (-0.067, 0.153)	0.065 (-0.146, 0.275)	**-0.209 (-0.409, -0.008)**	0.111 (-0.092, 0.314)
0.003 (-0.147, 0.152)	-0.024 (-0.148, 0.100)	**IJV index (%)**	**0.206 (0.048, 0.364)**	**0.195 (0.038, 0.352)**	0.071 (-0.057, 0.200)	0.019 (-0.124, 0.161)	0.040 (-0.192, 0.272)	**-0.233 (-0.450, -0.016)**	0.087 (-0.132, 0.306)
**-0.204 (-0.371, -0.037)**	**-0.231 (-0.383, -0.079)**	**-0.206 (-0.364, -0.048)**	**IJVmax (mm)**	-0.011 (-0.181, 0.158)	-0.135 (-0.284, 0.014)	**-0.188 (-0.350, -0.026)**	-0.166 (-0.411, 0.079)	**-0.440 (-0.669, -0.210)**	-0.120 (-0.351, 0.112)
**-0.193 (-0.359, -0.026)**	**-0.220 (-0.370, -0.069)**	**-0.195 (-0.352, -0.038)**	0.011 (-0.158, 0.181)	**IJVmin (mm)**	-0.124 (-0.271, 0.024)	**-0.176 (-0.338, -0.016)**	-0.155 (-0.399, 0.090)	**-0.428 (-0.657, -0.199)**	-0.108 (-0.339, 0.123)
-0.069 (-0.185, 0.047)	**-0.096 (-0.190, -0.002)**	-0.071 (-0.200, 0.057)	0.135 (-0.014, 0.284)	0.124 (-0.024, 0.271)	**IVCmax (mm)**	-0.053 (-0.160, 0.055)	-0.031 (-0.241, 0.178)	**-0.304 (-0.490, -0.119)**	0.016 (-0.173, 0.204)
-0.016 (-0.140, 0.108)	-0.043 (-0.153, 0.067)	-0.019 (-0.161, 0.124)	**0.188 (0.026, 0.350)**	0.176 (0.016, 0.338)	0.053 (-0.055, 0.160)	**IVCmin (mm)**	0.022 (-0.190, 0.233)	**-0.252 (-0.454, -0.049)**	0.068 (-0.137, 0.274)
-0.038 (-0.254, 0.178)	-0.065 (-0.275, 0.146)	-0.040 (-0.272, 0.192)	**0.166 (-0.079, 0.411)**	0.155 (-0.090, 0.399)	0.031 (-0.178, 0.241)	-0.022 (-0.233, 0.190)	**ΔIVC (mm)**	**-0.273 (-0.543, -0.003)**	0.047 (-0.225, 0.319)
**0.236 (0.050, 0.421)**	**0.209 (0.008, 0.409)**	**0.233 (0.016, 0.450)**	**0.440 (0.210, 0.669)**	**0.428 (0.199, 0.657)**	**0.304 (0.119, 0.490)**	**0.252 (0.049, 0.454)**	**0.273 (0.003, 0.543)**	**ΔCaval index (%)**	0.320 (0.116, 0.524)
-0.084 (-0.273, 0.104)	-0.111 (-0.314, 0.092)	-0.087 (-0.306, 0.132)	0.120 (-0.112, 0.351)	0.108 (-0.123, 0.339)	-0.016 (-0.204, 0.173)	-0.068 (-0.274, 0.137)	-0.047 (-0.319, 0.225)	**-0.320 (-0.524, -0.116)**	**ΔIVCDV (mm)**

Abbreviations: IVCD, inferior vena cava diameter; IVC, inferior vena cava; IJV, internal jugular vein diameter; IVCDV, inferior vena cava diameter variation; Spont, spontaneous; MV, mechanical ventilation.

Table S10. Meta-regression.

Association between Caval index (%) and fluid responsiveness defined by FC				
Variables	Studies, N	Univariate analysis		
		Coeff	SE	p value
Gold standard criterion	9	0.003	0.003	0.362
% of responders	8	0.004	0.010	0.683
Test criterion (cut-off)	6	-0.001	0.006	0.907
Mean age	8	0.004	0.016	0.809
Men, %	8	-0.009	0.007	0.211

Abbreviations: SE, standard error; FC, fluid challenge.

Meta-regression allows identification of which characteristics of the study could affect the results of the meta-analysis (effect size). In the context of our study, meta-regression was used to identify which characteristics of the studies (study-level variations) could affect the association between the Caval index (%) and fluid responsiveness defined by FC. Meta-regression can be either univariate or multivariate. All covariates (study characteristics) were tested only in the univariate model due to the large amount of missing data.

For a correct meta-regression, the Cochrane handbook recommends the use of a minimum of 10 studies for each study-level variable without providing justification, and there should be at least 6 to 10 studies for a continuous study-level variable. For a categorical subgroup variable, each subgroup should have a minimum of 4 studies.

Table S11. The estimated values of between-study variance and incoherence for the network meta-analysis.

This table presents the results of the between-study variance and incoherence estimation (consistency between direct and indirect comparisons)

Outcome	Parameter	Stated clinically important size of effect
Area under ROC curve	The estimated value of between-study variance $\tau 2$ (Bayesian method) 0.009†	0.1
Area under ROC curve	$\chi 2$ statistic: 68.321 (12 degrees of freedom), P value: <0.001#	0.1

†Heterogeneity is non-significant (relative to the clinically important size of effect)

#Incoherece is significant

Table S12. Per-comparison contribution matrix.

A per-comparison contribution matrix quantifies the relative influence of each direct and indirect comparison on the overall network meta-analysis estimates.

random MD	Caval index (%):IJV index (%)	Caval index (%):IJVmax (mm)	Caval index (%):IJVmin (mm)	Caval index (%):IVCmax (mm)	Caval index (%):IVCmin (mm)	Caval index (%):ΔIVC (mm)	Caval index (%):ΔCaval index (%)	Caval index (%):ΔIVCDV (mm)	IJV index (%):IJVmax (mm)	IJV index (%):IJVmin (mm)	IJV index (%):IVCmax (mm)	IJV index (%):IVCmin (mm)	IJVmax (mm):IJVmin (mm)	IJVmax (mm):IVCmax (mm)	IJVmax (mm):IVCmin (mm)	IJVmin (mm):IVCmax (mm)	IJVmin (mm):IVCmin (mm)	IVCmax (mm):IVCmin (mm)	IVCmax (mm): ΔIVC (mm)	IVCmax (mm): ΔCaval index (%)	IVCmax (mm): ΔIVCDV (mm)	IVCmin (mm): ΔIVC (mm)	ΔCaval index (%):ΔIVCDV (mm)
**Mixed estimates**																							
Caval index (%):IJV index (%)	41.12	4.83	4.91	10.47	5.59	0.35	0.48	0.47	6.52	6.41	9.79	4.39	0	1.11	0.58	0.92	0.58	0.17	0.22	0.48	0.47	0.14	0
Caval index (%):IJVmax (mm)	7.99	29.94	5.42	11.66	6.08	0.36	0.53	0.5	9.32	0.17	0.91	0.6	7.3	11.16	4.76	1.06	0.65	0.2	0.24	0.53	0.5	0.13	0
Caval index (%):IJVmin (mm)	7.99	5.32	30.17	11.68	6.04	0.36	0.53	0.5	0.17	9.32	0.91	0.6	7.18	1.04	0.65	11.22	4.71	0.21	0.24	0.53	0.5	0.12	0
Caval index (%):IVCmax (mm)	4.47	3.12	3.18	52.02	6.99	1.39	2.32	2.25	0.19	0.19	4.06	0.03	0	3.36	0.05	3.42	0.05	6.87	1.44	2.32	2.25	0.06	0
Caval index (%):IVCmin (mm)	3.95	2.83	2.87	11.23	49.76	1.95	0.67	0.58	0.13	0.11	0.24	3.48	0	0.04	2.92	0.05	2.93	12.52	0.28	0.67	0.58	2.22	0
Caval index (%):ΔCaval index (%)	1.48	1.04	0.97	13.01	1.75	0.35	39.92	7.33	0.06	0.06	1.35	0.01	0	1.11	0.01	1.04	0.01	1.72	0.36	16.1	2.5	0.02	9.82
Caval index (%):ΔIVCDV (mm)	1.48	1.04	0.94	13.01	1.75	0.35	7.56	39.45	0.06	0.06	1.35	0.01	0	1.11	0.01	1.01	0.01	1.72	0.36	2.58	15.99	0.02	10.13
Caval index (%):ΔIVC (mm)	1.89	1.3	1.32	12.08	10.65	36.59	0.54	0.52	0.09	0.07	0.94	0.79	0	0.76	0.62	0.74	0.65	1.12	14.46	0.54	0.52	13.83	0
IJV index (%):IJVmax (mm)	11.67	10.15	0.34	0.73	0.34	0.03	0.04	0.04	35.36	8.2	8.05	3.5	8.66	8.85	3.76	0.08	0.05	0.04	0.03	0.04	0.04	0.01	0
IJV index (%):IJVmin (mm)	11.68	0.33	10.18	0.73	0.32	0.03	0.04	0.04	8.07	35.63	8.05	3.5	8.53	0.08	0.06	8.86	3.72	0.05	0.03	0.04	0.04	0.01	0
IJV index (%):IVCmax (mm)	15.65	0.85	0.85	15.2	0.51	0.3	0.68	0.66	6.74	6.79	27.69	4.44	0	5.36	0.54	5.45	0.49	5.77	0.51	0.68	0.66	0.21	0
IJV index (%):IVCmin (mm)	16.27	1.33	1.03	0.16	17.87	0.58	0.01	0.01	6.48	6.13	10.14	15.74	0	1.32	3.83	1.26	3.84	12.44	0.46	0.01	0.01	1.05	0
IJVmax (mm):IJVmin (mm)	0	9.65	9.66	0	0.01	0	0	0	9.62	9.62	0	0	35.75	9.09	3.76	9.1	3.73	0.01	0	0	0	0	0
IJVmax (mm):IVCmax (mm)	1.99	11.85	1.11	13.35	0.09	0.27	0.63	0.61	8.34	0.07	5.8	0.63	7.4	29.27	4.69	5.65	0.57	5.79	0.46	0.63	0.61	0.19	0
IJVmax (mm):IVCmin (mm)	2.46	12.15	1.65	0.27	16.12	0.44	0.02	0.02	8.04	0.09	1.29	4.37	6.84	11.27	15.43	1.16	3.94	12.88	0.54	0.02	0.02	0.98	0
IJVmin (mm):IVCmax (mm)	1.98	1.09	11.91	13.37	0.11	0.27	0.63	0.61	0.07	8.34	5.8	0.63	7.29	5.55	0.58	29.51	4.64	5.76	0.46	0.63	0.61	0.19	0
IJVmin (mm):IVCmin (mm)	2.46	1.62	12.22	0.27	16.16	0.44	0.02	0.02	0.08	8.06	1.3	4.38	6.76	1.13	3.94	11.34	15.34	12.92	0.54	0.02	0.02	0.98	0
IVCmax (mm):IVCmin (mm)	0.23	0.2	0.21	14.79	16.82	0.27	0.88	0.78	0.02	0.03	3.61	3.43	0	3.24	3.03	3.27	3.04	40.06	2.09	0.88	0.78	2.36	0
IVCmax (mm):ΔCaval index (%)	1.48	1.04	0.97	13.01	1.75	0.35	16.1	2.5	0.06	0.06	1.35	0.01	0	1.11	0.01	1.04	0.01	1.72	0.36	39.92	7.33	0.02	9.82
IVCmax (mm):ΔIVCDV (mm)	1.48	1.04	0.94	13.01	1.75	0.35	2.58	15.99	0.06	0.06	1.35	0.01	0	1.11	0.01	1.01	0.01	1.72	0.36	7.56	39.45	0.02	10.13
IVCmax (mm):ΔIVC (mm)	1.16	0.78	0.61	13.94	1.8	15.92	0.62	0.6	0.06	0.06	1.77	0.72	0	1.54	0.7	1.37	0.7	9.13	34.28	0.62	0.6	13.04	0
IVCmin (mm):ΔIVC (mm)	0.9	0.56	0.43	0.43	14.24	15.64	0.03	0.03	0.03	0.03	0.68	1.53	0	0.69	1.29	0.7	1.15	10.91	13.46	0.03	0.03	37.21	0
ΔCaval index (%):ΔIVCDV (mm)	0	0	0	0	0	0	12.4	12.4	0	0	0	0	0	0	0	0	0	0	0	12.4	12.4	0	50.39
**Indirect estimates**																							
IJV index (%):ΔCaval index (%)	18.22	1.76	1.66	0.78	1.05	0.02	18.74	3.2	4.45	4.3	11.82	2.13	0	2.41	0.28	2.37	0.28	1.5	0.11	16.34	2.64	0.13	5.83
IJV index (%):ΔIVCDV (mm)	18.22	1.73	1.66	0.78	1.05	0.02	3.3	18.6	4.42	4.27	11.82	2.13	0	2.41	0.28	2.34	0.28	1.5	0.11	2.73	16.22	0.13	6.03
IJV index (%):ΔIVC (mm)	17.59	1.44	1.31	0.53	2.22	17.52	0.04	0.04	4.68	4.38	11.2	5.58	0	2.01	1.23	1.83	1.23	1.19	14.46	0.04	0.04	11.45	0
IJVmax (mm):ΔCaval index (%)	3.73	13.41	1.91	0.35	1.47	0.04	17.07	3.15	6.46	0.09	2.51	0.31	4.6	12.9	2.36	2.28	0.32	1.4	0.08	16.77	2.76	0.12	5.9
IJVmax (mm):ΔIVCDV (mm)	3.7	13.41	1.91	0.35	1.47	0.04	3.25	16.94	6.43	0.09	2.51	0.31	4.57	12.9	2.36	2.25	0.32	1.4	0.08	2.85	16.64	0.12	6.1
IJVmax (mm):ΔIVC (mm)	3.21	13.03	1.72	0.05	1.92	15.97	0	0	6.58	0.12	2.1	1.39	4.89	12.31	5.74	1.75	1.3	1.36	14.86	0	0	11.71	0
IJVmin (mm):ΔCaval index (%)	3.72	1.88	13.5	0.35	1.46	0.04	17.1	3.15	0.09	6.46	2.51	0.32	4.52	2.23	0.32	12.99	2.34	1.39	0.08	16.8	2.75	0.12	5.9
IJVmin (mm):ΔIVCDV (mm)	3.69	1.88	13.5	0.35	1.46	0.04	3.25	16.97	0.09	6.43	2.51	0.32	4.49	2.2	0.32	12.99	2.34	1.39	0.08	2.85	16.67	0.12	6.09
IJVmin (mm):ΔIVC (mm)	3.2	1.69	13.11	0.05	1.93	16	0	0	0.11	6.57	2.1	1.39	4.82	1.71	1.31	12.38	5.69	1.37	14.88	0	0	11.68	0
IVCmin (mm):ΔCaval index (%)	0.94	0.63	0.64	0.8	20.56	0.5	18.96	3.52	0.03	0.02	1.12	2.01	0	0.82	1.48	0.83	1.49	16.6	0.68	17.96	2.87	1.18	6.38
IVCmin (mm):ΔIVCDV (mm)	0.94	0.63	0.62	0.75	20.49	0.5	3.68	18.76	0.03	0.02	1.12	2.01	0	0.82	1.48	0.83	1.47	16.6	0.68	2.95	17.83	1.18	6.63
ΔIVC (mm):ΔCaval index (%)	0.23	0.13	0.13	0.31	3.77	17.6	18.45	3.1	0.01	0.01	0.25	0.46	0	0.24	0.37	0.24	0.38	3.23	16.4	17.94	2.72	8.21	5.81
ΔIVC (mm):ΔIVCDV (mm)	0.23	0.13	0.13	0.31	3.74	17.6	3.2	18.32	0.01	0.01	0.25	0.46	0	0.24	0.37	0.24	0.38	3.2	16.4	2.81	17.82	8.15	6.01

Abbreviations: IVCD, inferior vena cava diameter; IVC, inferior vena cava; IJV, internal jugular vein diameter; IVCDV, inferior vena cava diameter variation. Columns refer to comparisons with direct data and rows refer to NMA relative comparisons.

Table S13. Percentage contribution matrix.

A percentage contribution matrix illustrates the proportional impact of each study or comparison on the final estimates in a network meta-analysis.

random MD	Ismail M., 2022	Doucet J.J., 2020	Bortolotti P., 2018	Taccheri T., 2021	Ma G.G., 2018	Baker A.K., 2013	Elsaeed A., 2022	Airapetian N., 2015	Ma Q., 2022
**Mixed estimates**									
Caval index (%):IJV index (%)	2.88	37.7	2.79	3.73	30.63	1.11	15.99	2.34	2.82
Caval index (%):IJVmax (mm)	3.18	47.48	3.07	4.1	32.19	1.21	3.11	2.6	3.06
Caval index (%):IJVmin (mm)	3.17	48.38	3.06	4.11	31.3	1.2	3.11	2.61	3.06
Caval index (%):IVCmax (mm)	11.05	22.14	10.36	18.21	12.37	1.39	1.74	11.62	11.11
Caval index (%):IVCmin (mm)	17.43	7.44	18.35	4.46	20.61	9.9	1.54	2.51	17.76
Caval index (%):ΔCaval index (%)	2.76	6.5	2.59	77.93	3.6	0.35	0.57	2.9	2.79
Caval index (%):ΔIVCDV (mm)	2.76	6.46	2.59	77.97	3.59	0.35	0.57	2.9	2.79
Caval index (%):ΔIVC (mm)	4.62	6.2	4.6	4.23	6.49	2.12	0.73	2.7	68.31
IJV index (%):IJVmax (mm)	0.2	52.52	0.19	0.29	41.82	0.07	4.54	0.16	0.21
IJV index (%):IJVmin (mm)	0.2	53.58	0.19	0.29	40.76	0.06	4.54	0.16	0.21
IJV index (%):IVCmax (mm)	3.98	41.8	3.83	5.32	31.4	0.1	6.09	3.39	4.08
IJV index (%):IVCmin (mm)	8.09	22.65	8.66	0.07	42.14	3.55	6.33	0.04	8.46
IJVmax (mm):IJVmin (mm)	0.01	60.44	0.01	0	39.53	0	0	0	0.01
IJVmax (mm):IVCmax (mm)	3.63	49.42	3.5	4.81	31.13	0.02	0.77	2.98	3.72
IJVmax (mm):IVCmin (mm)	7.81	28.66	8.35	0.11	42.7	3.21	0.96	0.06	8.13
IJVmin (mm):IVCmax (mm)	3.62	50.34	3.5	4.81	30.24	0.02	0.77	2.98	3.72
IJVmin (mm):IVCmin (mm)	7.83	29.26	8.38	0.12	42.04	3.21	0.96	0.06	8.15
IVCmax (mm):IVCmin (mm)	18.14	9.78	19	5.9	21.23	3.34	0.09	3.3	19.22
IVCmax (mm):ΔCaval index (%)	2.76	6.5	2.59	77.93	3.6	0.35	0.57	2.9	2.79
IVCmax (mm):ΔIVCDV (mm)	2.76	6.46	2.59	77.97	3.59	0.35	0.57	2.9	2.79
IVCmax (mm):ΔIVC (mm)	5.12	6.92	5.09	4.88	6.83	0.36	0.45	3.11	67.24
IVCmin (mm):ΔIVC (mm)	6.78	2.5	7.25	0.19	8.32	2.83	0.35	0.09	71.66
ΔCaval index (%):ΔIVCDV (mm)	0	0	0	99.99	0	0	0	0	0
**Indirect estimates**									
IJV index (%):ΔCaval index (%)	0.81	24.58	0.85	46.88	18.51	0.21	7.09	0.17	0.9
IJV index (%):ΔIVCDV (mm)	0.81	24.5	0.85	47.01	18.46	0.21	7.09	0.17	0.9
IJV index (%):ΔIVC (mm)	0.97	22.99	1.02	0.24	23.2	0.44	6.84	0.12	44.17
IJVmax (mm):ΔCaval index (%)	0.83	30.5	0.87	45.7	19.38	0.29	1.45	0.08	0.89
IJVmax (mm):ΔIVCDV (mm)	0.83	30.43	0.87	45.83	19.34	0.29	1.44	0.08	0.89
IJVmax (mm):ΔIVC (mm)	0.88	29.09	0.94	0.02	24.19	0.38	1.25	0.01	43.22
IJVmin (mm):ΔCaval index (%)	0.82	31.08	0.87	45.75	18.78	0.29	1.45	0.08	0.88
IJVmin (mm):ΔIVCDV (mm)	0.82	31	0.87	45.88	18.73	0.29	1.43	0.08	0.88
IJVmin (mm):ΔIVC (mm)	0.89	29.66	0.95	0.02	23.57	0.38	1.24	0.01	43.26
IVCmin (mm):ΔCaval index (%)	10.08	3.22	10.76	49.83	11.17	4.09	0.37	0.18	10.3
IVCmin (mm):ΔIVCDV (mm)	10.05	3.19	10.74	49.97	11.14	4.08	0.37	0.17	10.28
ΔIVC (mm):ΔCaval index (%)	1.93	0.81	2.05	48.07	2.47	0.75	0.09	0.07	43.73
ΔIVC (mm):ΔIVCDV (mm)	1.91	0.81	2.04	48.2	2.46	0.74	0.09	0.07	43.65

Abbreviations: IVCD, inferior vena cava diameter; IVC, inferior vena cava; IJV, internal jugular vein diameter; IVCDV, inferior vena cava diameter variation. The rows refer to the studies, and columns refer to the comparisons (grouped into mixed and indirect estimates) from network meta-analysis. The entries show how much each study contributes (as percentage) to the estimation of the mean difference for comparisons. Mixed comparisons are estimated using both direct and indirect evidence.

Table S14. Risk of bias analysis.

№	Study	Test parameter	Test cutoff	Risk of bias (QUADAS-2)				OVERALL bias	Applicability concerns (QUADAS-2)		
				P	I	R	FT		P	I	R
1	Airapetian N.	Caval index (%) Spont	42	✓	✓	✓	✓	Low	✓	✓	✓
1	Airapetian N.	IVCmax (mm)	21.0	✓	✓	✓	✓	Low	✓	✓	✓
2	Ismail M.	Caval index (%)	40.0	✓	✓	✗	✓	Moderate	✓	✓	✓
2	Ismail M.	IVCmax (mm)	13.0	✓	✓	✗	✓	Moderate	✓	✓	✓
2	Ismail M.	IVCmin (mm)	5.0	✓	✓	✗	✓	Moderate	✓	✓	✓
3	Doucet J.J.	Caval index (%) Spont	ND	✗	✗	✗	✓	High	✓	✓	✓
3	Doucet J.J.	IJV index (%)	ND	✗	✗	✗	✓	High	✓	✓	✓
3	Doucet J.J.	IJVmax (mm)	ND	✗	✗	✗	✓	High	✓	✓	✓
3	Doucet J.J.	IJVmin (mm)	ND	✗	✗	✗	✓	High	✓	✓	✓
3	Doucet J.J.	IVCmax (mm)	ND	✗	✗	✗	✓	High	✓	✓	✓
4	Elsaeed A.	Caval index (%) Spont	35.0	✓	✓	✓	✓	Low	✓	✓	✓
4	Elsaeed A.	IJV index (%)	17.56	✓	✓	✓	✓	Low	✓	✓	✓
5	Bortolotti P.	Caval index (%) Spont	39	✓	✓	✓	✓	Low	✓	✓	✓
5	Bortolotti P.	IVCmax (mm)	18	✓	✓	✓	✓	Low	✓	✓	✓
5	Bortolotti P.	IVCmin (mm)	11	✓	✓	✓	✓	Low	✓	✓	✓
6	Taccheri T.	Caval index (%) MV	ND	✓	✗	✓	✓	Moderate	✓	✓	✓
6	Taccheri T.	IVCmax (mm)	ND	✓	✗	✓	✓	Moderate	✓	✓	✓
6	Taccheri T.	ΔCaval index (%)	4	✓	✗	✓	✓	Moderate	✓	✓	✓
6	Taccheri T.	ΔIVCDV (mm)	ND	✓	✗	✓	✓	Moderate	✓	✓	✓
7	Ma G.G.	Caval index (%) MV	13.39	✓	✓	✓	✓	Low	✓	✓	✓
7	Ma G.G.	IJV index (%)	12.99	✓	✓	✓	✓	Low	✓	✓	✓
7	Ma G.G.	IJVmax (mm)	8.6	✓	✓	✓	✓	Low	✓	✓	✓
7	Ma G.G.	IJVmin (mm)	6.4	✓	✓	✓	✓	Low	✓	✓	✓
7	Ma G.G.	IVCmax (mm)	15.7	✓	✓	✓	✓	Low	✓	✓	✓
7	Ma G.G.	IVCmin (mm)	14	✓	✓	✓	✓	Low	✓	✓	✓
8	Baker A.K.	Caval index (%) MV	ND	✓	✗	✓	✓	Moderate	✓	✓	✓
8	Baker A.K.	IVCmin (mm)	ND	✓	✗	✓	✓	Moderate	✓	✓	✓
9	Ma Q.	ΔIVC (mm)	2.9	✓	✓	✓	✓	Low	✓	✓	✓
9	Ma Q.	Caval index (%) MV	15.32	✓	✓	✓	✓	Low	✓	✓	✓
9	Ma Q.	Caval index (%) Spont	30.25	✓	✓	✓	✓	Low	✓	✓	✓
9	Ma Q.	IVCmax (mm)	15.3	✓	✓	✓	✓	Low	✓	✓	✓
9	Ma Q.	IVCmin (mm)	11.4	✓	✓	✓	✓	Low	✓	✓	✓

P = patient selection; I = index test; R = reference standard; FT = flow and timing.
✓ indicates low risk; ✗ indicates high risk; ? indicates unclear risk.

Figure S1. SUCRA radial plot and ranking values.

A SUCRA radial plot and ranking values represent the relative effectiveness of interventions, based on their cumulative ranking probabilities in a network meta-analysis.

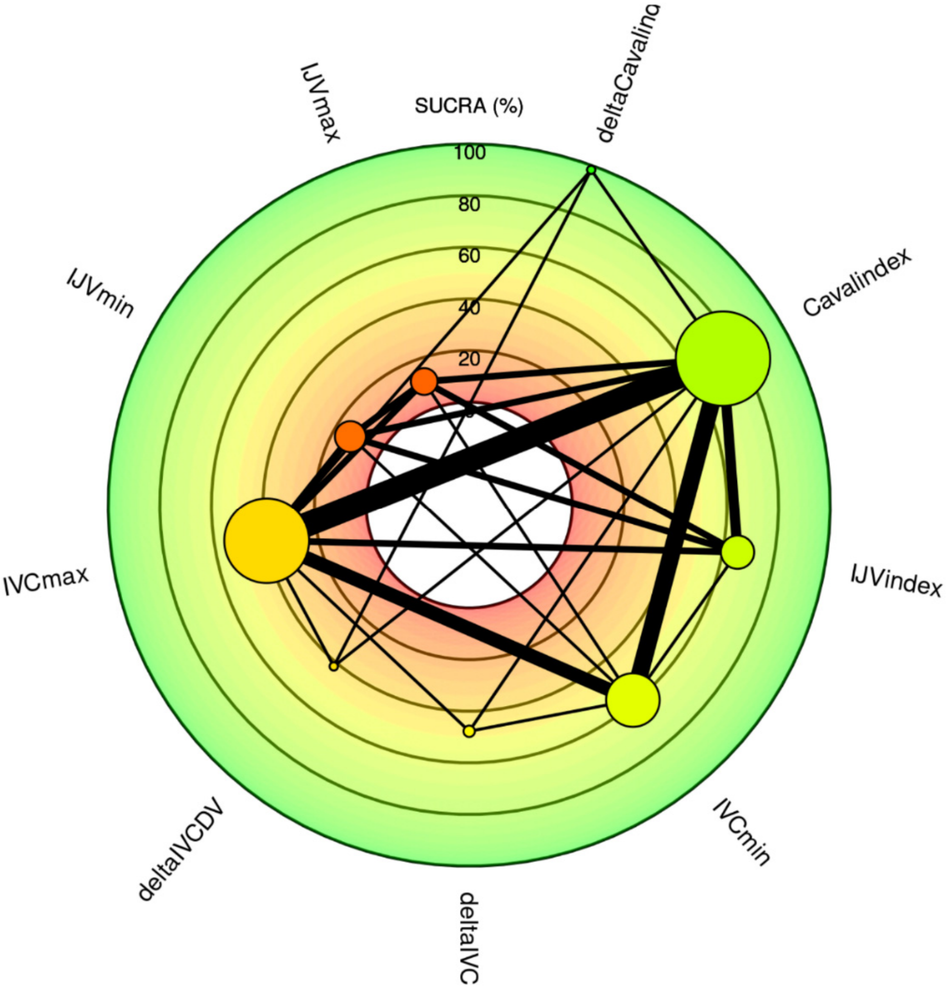

Treatment	Rank 1	Rank 2	Rank 3	Rank 4	Rank 5	Rank 6	Rank 7	Rank 8	Rank 9	SUCRA
Cavalindex	0.01	0.31	0.37	0.21	0.08	0.02	0.00	0.00	0.00	73.67
deltaCavalindex	0.93	0.04	0.01	0.01	0.00	0.00	0.00	0.00	0.00	98.35
deltaIVC	0.03	0.17	0.10	0.11	0.12	0.14	0.17	0.09	0.08	47.75
deltaIVCDV	0.01	0.11	0.08	0.10	0.12	0.16	0.21	0.11	0.09	41.73
IJVindex	0.02	0.25	0.22	0.20	0.15	0.10	0.05	0.01	0.00	65.69
IJVmax	0.00	0.00	0.00	0.01	0.02	0.04	0.12	0.34	0.45	11.05
IJVmin	0.00	0.00	0.01	0.01	0.03	0.05	0.15	0.38	0.37	13.22
IVCmax	0.00	0.00	0.02	0.09	0.24	0.36	0.23	0.04	0.01	39.76
IVCmin	0.01	0.11	0.19	0.26	0.23	0.13	0.06	0.01	0.00	58.78

Figure S2. Risk of bias bar chart for studied comparisons.

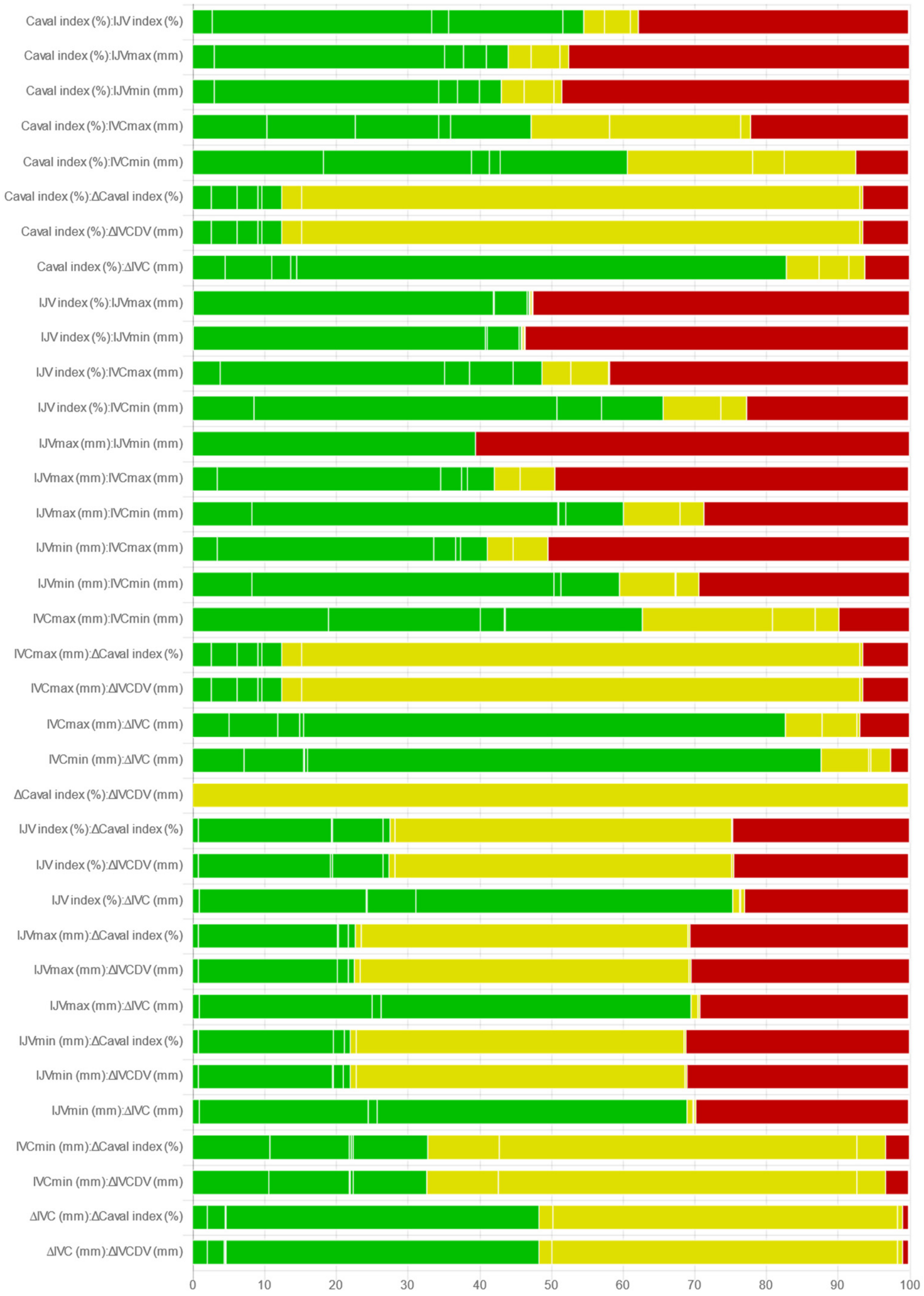

Abbreviations: IVCD, inferior vena cava diameter; IVC, inferior vena cava; IJV, internal jugular vein diameter; IVCDV, inferior vena cava diameter variation.

White vertical lines separate the percentage contribution of different studies. Each bar shows the percentage contribution from studies judged to be at low (green), moderate (yellow) and high (red) risk of bias.

Figure S3. Network meta-analysis regression plot.

A network meta-analysis regression plot visually depicts the relationship between study-level covariates and treatment effects, helping to assess potential effect modifiers and heterogeneity.

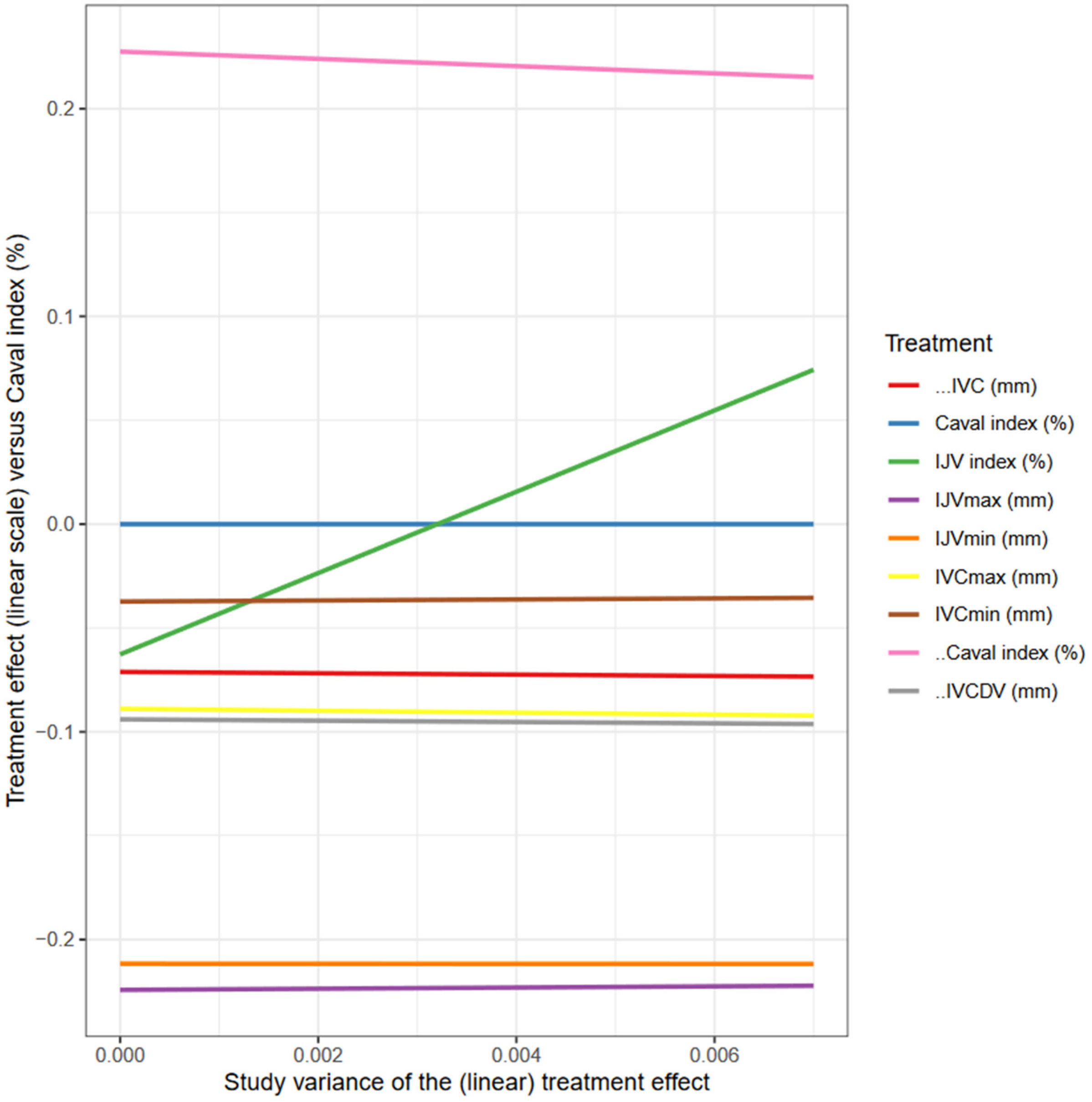

Abbreviations: IVCD, inferior vena cava diameter; IVC, inferior vena cava; IJV, internal jugular vein diameter; IVCDV, inferior vena cava diameter variation.

Figure S4. Certainty of evidence assessment (CINeMA approach).

Certainty of evidence assessment using the CINeMA approach evaluates confidence in network meta-analysis results by considering six domains—within-study bias, reporting bias, indirectness, imprecision, heterogeneity, and incoherence.

Comparison	Number of Studies	Within-study bias	Reporting bias	Indirectness	Imprecision	Heterogeneity	Incoherence	Confidence rating	Reason(s) for downgrading
**Mixed evidence**									
Caval index (%) MV vs Caval index (%) Spont	1	Some concerns ☐	Low risk	No concerns	Some concerns ☑	No concerns	No concerns	Moderate	| Imprecision |
Caval index (%) vs IJV index (%)	3	Some concerns ☐	Low risk	No concerns	Some concerns ☐	Some concerns ☑	Major concerns ☑	Very low	| Heterogeneity | Incoherence |
Caval index (%) vs IJVmax (mm)	2	Some concerns ☑	Low risk	No concerns	No concerns	Some concerns ☑	Major concerns ☑	Very low	| Within-study bias | Heterogeneity | Incoherence |
Caval index (%) vs IJVmin (mm)	2	Some concerns ☑	Low risk	No concerns	No concerns	Some concerns ☑	Major concerns ☑	Very low	| Within-study bias | Heterogeneity | Incoherence |
Caval index (%) vs IVCmax (mm)	7	Some concerns ☐	Low risk	No concerns	No concerns	Major concerns ☑	No concerns	Low	| Heterogeneity |
Caval index (%) vs IVCmin (mm)	5	No concerns	Low risk	No concerns	Some concerns ☐	Some concerns ☑	No concerns	Moderate	| Heterogeneity |
Caval index (%) vs ΔCaval index (%)	1	Some concerns ☐	Low risk	No concerns	No concerns	Some concerns ☑	No concerns	Moderate	| Heterogeneity |
Caval index (%) vs ΔIVCDV (mm)	1	Some concerns ☐	Low risk	No concerns	Some concerns ☐	Some concerns ☑	No concerns	Moderate	| Heterogeneity |
Caval index (%) vs ΔIVC (mm)	1	No concerns	Low risk	No concerns	Major concerns ☑	No concerns	No concerns	Low	| Imprecision |
IJV index (%) vs IJVmax (mm)	2	Some concerns ☐	Low risk	No concerns	No concerns	Some concerns ☑	No concerns	Moderate	| Heterogeneity |
IJV index (%) vs IJVmin (mm)	2	Some concerns ☐	Low risk	No concerns	No concerns	Some concerns ☑	No concerns	Moderate	| Heterogeneity |
IJV index (%) vs IVCmax (mm)	2	Some concerns ☑	Low risk	No concerns	Some concerns ☐	Some concerns ☑	No concerns	Low	| Within-study bias | Heterogeneity |
IJV index (%) vs IVCmin (mm)	1	Some concerns ☐	Low risk	No concerns	Major concerns ☑	No concerns	Major concerns ☑	Very low	| Imprecision | Incoherence |
IJVmax (mm) vs IJVmin (mm)	2	Some concerns ☐	Low risk	No concerns	Major concerns ☑	No concerns	Major concerns ☑	Very low	| Imprecision | Incoherence |
IJVmax (mm) vs IVCmax (mm)	2	Some concerns ☐	Low risk	No concerns	Some concerns ☐	Some concerns ☑	No concerns	Moderate	| Heterogeneity |
IJVmax (mm) vs IVCmin (mm)	1	Some concerns ☑	Low risk	No concerns	No concerns	Some concerns ☑	No concerns	Low	| Within-study bias | Heterogeneity |
IJVmin (mm) vs IVCmax (mm)	2	Some concerns ☐	Low risk	No concerns	Some concerns ☐	Some concerns ☑	No concerns	Moderate	| Heterogeneity |
IJVmin (mm) vs IVCmin (mm)	1	Some concerns ☑	Low risk	No concerns	No concerns	Some concerns ☑	No concerns	Low	| Within-study bias | Heterogeneity |
IVCmax (mm) vs IVCmin (mm)	4	No concerns	Low risk	No concerns	Some concerns ☐	Some concerns ☑	No concerns	Moderate	| Heterogeneity |
IVCmax (mm) vs ΔCaval index (%)	1	Some concerns ☐	Low risk	No concerns	No concerns	No concerns	No concerns	High	|
IVCmax (mm) vs ΔIVCDV (mm)	1	Some concerns ☐	Low risk	No concerns	Major concerns ☑	No concerns	No concerns	Low	| Imprecision |
IVCmax (mm) vs ΔIVC (mm)	1	No concerns	Low risk	No concerns	Major concerns ☑	No concerns	No concerns	Low	| Imprecision |
IVCmin (mm) vs ΔIVC (mm)	1	No concerns	Low risk	No concerns	Major concerns ☑	No concerns	No concerns	Low	| Imprecision |
ΔCaval index (%) vs ΔIVCDV (mm)	1	Some concerns ☐	Low risk	No concerns	No concerns	No concerns	Major concerns ☑	Low	| Incoherence |
**Indirect evidence**									
IJV index (%) vs ΔCaval index (%)	--	Some concerns ☑	Low risk	No concerns	No concerns	Some concerns ☑	Major concerns ☑	Very low	| Within-study bias | Heterogeneity | Incoherence |
IJV index (%) vs ΔIVCDV (mm)	--	Some concerns ☑	Low risk	No concerns	Major concerns ☑	No concerns	Major concerns ☑	Very low	| Within-study bias | Imprecision | Incoherence |
IJV index (%) vs ΔIVC (mm)	--	No concerns	Low risk	No concerns	Major concerns ☑	No concerns	Major concerns ☑	Very low	| Imprecision | Incoherence |
IJVmax (mm) vs ΔCaval index (%)	--	Some concerns ☐	Low risk	No concerns	No concerns	No concerns	Major concerns ☑	Low	| Incoherence |
IJVmax (mm) vs ΔIVCDV (mm)	--	Some concerns ☐	Low risk	No concerns	Some concerns ☐	Some concerns ☑	Major concerns ☑	Very low	| Heterogeneity | Incoherence |
IJVmax (mm) vs ΔIVC (mm)	--	Some concerns ☐	Low risk	No concerns	Some concerns ☐	Some concerns ☑	Major concerns ☑	Very low	| Heterogeneity | Incoherence |
IJVmin (mm) vs ΔCaval index (%)	--	Some concerns ☐	Low risk	No concerns	No concerns	No concerns	Major concerns ☑	Low	| Incoherence |
IJVmin (mm) vs ΔIVCDV (mm)	--	Some concerns ☐	Low risk	No concerns	Some concerns ☐	Some concerns ☑	Major concerns ☑	Very low	| Heterogeneity | Incoherence |
IJVmin (mm) vs ΔIVC (mm)	--	Some concerns ☐	Low risk	No concerns	Some concerns ☐	Some concerns ☑	Major concerns ☑	Very low	| Heterogeneity | Incoherence |
IVCmin (mm) vs ΔCaval index (%)	--	Some concerns ☐	Low risk	No concerns	No concerns	Some concerns ☑	Major concerns ☑	Very low	| Heterogeneity | Incoherence |
IVCmin (mm) vs ΔIVCDV (mm)	--	Some concerns ☐	Low risk	No concerns	Major concerns ☑	No concerns	Major concerns ☑	Very low	| Imprecision | Incoherence |
ΔCaval index (%) vs ΔIVC (mm)	--	Some concerns ☐	Low risk	No concerns	No concerns	Some concerns ☑	Major concerns ☑	Very low	| Heterogeneity | Incoherence |
ΔIVCDV (mm) vs ΔIVC (mm)	--	Some concerns ☐	Low risk	No concerns	Major concerns ☑	No concerns	Major concerns ☑	Very low	| Imprecision | Incoherence |

Abbreviations: IVCD, inferior vena cava diameter; IVC, inferior vena cava; IJV, internal jugular vein diameter; IVCDV, inferior vena cava diameter variation.
